# A Transdisciplinary Framework to Bridge Science–Policy–Development Gaps in Global Land Management Initiatives

**DOI:** 10.1002/gch2.202400261

**Published:** 2025-05-22

**Authors:** Nigussie Haregeweyn, Atsushi Tsunekawa, Ayele Almaw Fenta, Pasquale Borrelli, Panos Panagos, Ermias Aynekulu, Takeshi Abe, Peri Pablo, Simon West

**Affiliations:** ^1^ International Platform for Dryland Research and Education Tottori University 1390 Hamasaka Tottori 680‐0001 Japan; ^2^ Department of Environmental Sciences Environmental Geosciences University of Basel Basel 4056 Switzerland; ^3^ Department of Science Roma Tre University Rome 00154 Italy; ^4^ European Commission Joint Research Centre Ispra VA I‐21027 Italy; ^5^ World Agroforestry (ICRAF) UN Avenue Nairobi P.O. Box 30677 Kenya; ^6^ Instituto Nacional de Tecnología Agropecuaria (INTA) Santa Cruz Río Gallegos 9400 Argentina; ^7^ Crawford School of Public Policy The Australian National University Canberra 2601 Australia; ^8^ Stockholm Resilience Centre Stockholm University Stockholm SE‐10691 Sweden; ^9^ Northern Institute Charles Darwin University Darwin NT 0810 Australia

**Keywords:** desertification, science–practice interface, science–policy–development interface, soil erosion, sustainable land management

## Abstract

Effective implementation of Sustainable Land Management (SLM) remains a major challenge worldwide because of its weak integration within the domains of science, policy, and development practice. Based on global analyses of soil erosion risk and the degree of implementation of SLM research, policies, and practices at the country level, we propose a transdisciplinary framework to address soil erosion through SLM. In the analysis, we used indices of the policy–development, science–policy, and science–development interfaces to evaluate the overall science–policy–development interface (SPDI) in 236 countries. Over 190 countries (81%) were found to be currently facing moderate or high risk of increased soil erosion from two or more erosion processes, and 182 countries (77%) were found to have a SPDI level that was lower than their soil erosion risk implying the urgent need for a transdisciplinary framework that supports the implementation of future soil erosion research and development projects. Our proposed transdisciplinary framework comprises seven stages, starting from “shared research framing” and ending with “ex‐post evaluation”. The framework’s practical application is discussed in the context of a recent project, emphasizing the need for country‐specific studies to develop tailored frameworks.

## Graphical abstract

This study presents a transdisciplinary framework to enhance Sustainable Land Management (SLM) by strengthening the weak integration between science, policy, and development. A global analysis of 236 countries revealed widespread soil erosion risks and inadequate SLM implementation. The proposed seven‐stage framework aims to improve coordination across sectors and guide future research and policy interventions through country‐specific adaptations.

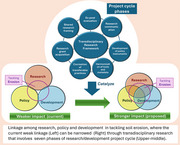

## 1 Introduction

Sustainable land management (SLM), which refers to the use of land resources while ensuring their long‐term productive potential, is being promoted worldwide as a means of tackling desertification, land degradation, and drought as part of the United Nations Convention to Combat Desertification.^[^ ^1^, ^2^ ^]^ SLM is promoted widely as a “triple win” strategy to enhance ecological health, land productivity, and human well‐being.^[^ ^3^ ^]^ The key features for a successful SLM according to the Food and Agriculture Organization of the United Nations (FAO)^[^ ^4^ ^]^ are as follows: 1) targeted policy and institutional support, 2) the integrated use of natural resources, and 3) multilevel, multi‐stakeholder involvement and partnerships at all levels. In these features, however, research evidence seems overlooked or not explicitly stated. The sustainability of development interventions such as SLM is often complex as they take place not only across the local, regional, and global scales but also at the point where science, policy, and development intersect. In the context of this study, **“**development**”** is defined as the practical application and implementation of SLM knowledge, strategies, and decisions in real‐world settings. It involves actions taken by various stakeholders, including farmers, to address challenges and effectively manage land resources. To conceptualize and visualize the science–policy–development interface (SPDI), various approaches have been developed;^[^ ^5^, ^6^, ^7^, ^8^, ^9^, ^10^ ^]^ however, there is currently no approach that provides a step‐by‐step plan for the implementation of SLM. In addition, the need for evidence‐based SLM dissemination and policy is often overlooked because science, policy, and development are often viewed as separate fields with distinct cultures, motivations, timelines, and incentives.^[^ ^9^, ^10^ ^]^

Several recent studies have identified areas for improvement with regard to the implementation of SLM practices. In a recent global‐scale review of the current progress of SLM initiatives, Haregeweyn et al.^[^ ^11^ ^]^ highlighted the lack of dissemination of evidence‐based SLM practices, particularly in developing countries that are already experiencing severe erosion rates. Similarly, in an interactive survey conducted at an FAO webinar on soil governance,^[^ ^12^ ^]^ 65% out of 160 participating countries reported that their country lacks specific soil laws that provide a legislative framework for developing policies and actions for sustainable soil management, highlighting the lack of SLM‐related policies at the global scale. A study by van Harena et al.,^[^ ^13^ ^]^ conducted on the basis of World Overview of Conservation Approaches and Technologies (WOCAT)’s global database, has shown that community‐based SLM initiatives are often overlooked by policymakers and government institutions, leading to insufficient institutional support, policies, economic incentives, and technical assistance. Traditionally, researchers often approach science uptake by policymakers and practitioners as a linear one‐way process, meaning that exploration of how policymakers and practitioners actually engage with and use research in policy and decision‐making is often overlooked.^[^ ^14^, ^15^ ^]^ Although several studies^[^ ^5^, ^7^, ^8^ ^]^ have emphasized knowledge co‐creation as a cornerstone within the sustainability sciences, Wyborn et al.^[^ ^16^ ^]^ have stressed that this activity must go beyond traditional stakeholder engagement through meetings and extend into the development of shared research framings and practices. Addressing sustainability challenges is complex, and as a challenge for sustainable development in the 21st century (UNDESA, 2015),^[^ ^17^ ^]^ it requires diverse expertise in combination with appropriate societal choices, policies, and practices.^[^ ^18^ ^]^ Therefore, how the three key factors of science, policy, and development interact could hamper scaling up and ultimately decide whether current global development initiatives including SLM are sustainable (e.g., Meilana et al., 2023).^[^ ^19^, ^20^ ^]^

The need for a holistic framework to address gaps in science, policy, and development linkages has been emphasized.^[^ ^9^ ^]^ Solving this issue will require integrated study to identify the missing linkages among the science, policy, and development aspects of SLM. There is a need for an integrated framework covering the scientific, policy, and developmental dimensions for tackling one of the greatest challenges of soil resources—soil erosion.^[^ ^19^, ^21^, ^22^, ^23^ ^]^ To this end, UNCCD Decision 3/COP.14 advocates for the integration of Sustainable Development Goal 15 and its related target 15.3 into the implementation of the UNCCD's land degradation neutrality principles.^[^ ^24^ ^]^ Recent land‐management initiatives, such as nature‐based solutions,^[^ ^25^ ^]^ call for a transdisciplinary research approach that links practitioners, policymakers, and scientists from different disciplines and which engages with citizens and other users.

Here, we assessed the links between science, policy, and development and SLM by analyzing to what extent countries that are at increased risks of future soil erosion have addressed SLM implementation from the viewpoint of evidence‐based dissemination of SLM practices, implementation of science‐based policy, and SLM‐directed policy development. Unlike traditional studies that focus primarily on science–policy (SP) or since–practice interactions, this paper adds a critical third component—development—evaluating these linkages in the context of actual soil erosion risks. By using the outcomes of this global assessment, we then propose a transdisciplinary framework to enhance the real‐world applicability of SLM by promoting a more collaborative, evidence‐based decision‐making process. Ultimately, our findings contribute to global efforts to achieve UNCCD's Target 15.3 of a land degradation‐neutral world by 2030 and beyond.^[^ ^3^ ^]^

## 2 Experimental Section

Integrated methods were employed using multiple datasets and a multicriteria framework to assess SLM in relation to research, policy, and development. The compiled data were studied from bibliometric sources, conservation databases, legal instruments, and soil erosion risk maps to construct indices reflecting the SPDI. These indices were mapped and analyzed using ArcGIS, with classification based on the variance minimization method. A transdisciplinary project cycle framework was also proposed to enhance collaboration among stakeholders.

### 2.1 Study Overview and Data Sources

To obtain a global picture of SLM research, development practice, policy, and erosion risk, a range of recognized country‐level data sources were identified. Generally, these sources were operated independently and currently without coordination or integration. While these sources can only provide a limited view of each field (e.g., scientific databases were used to assess the “research” field, which excludes local, practitioner, and Indigenous knowledge) they provide provisionally useful proxies to obtain a global overview. A total of four datasets were developed (Table S1, Supporting Information). The research and development practice data were compiled, analyzed, and published as part of the earlier research detailed in Haregeweyn et al.^[^ ^11^ ^]^

For the first dataset, to assess the progress of research related to SLM, articles indexed were searched in the Scopus (https://www.scopus.com) and Web of Science (https://login.webofknowledge.com) bibliometric databases by using search terms (TITLE‐ABS‐KEY (“soil erosion”) AND TITLE‐ABS‐KEY (“soil and water conservation”) OR TITLE‐ABS‐KEY (sustainable land management) AND DOCTYPE (article or review) AND PUBYEAR 1992 AND PUBYEAR 2020 AND EXCLUDE (LANGUAGE, all other than English)). This produced a dataset of 1151 SLM‐related scientific articles. Details on the dataset (e.g., inclusion/exclusion criteria for narrowing down the search results and ensuring the articles were relevant) can be found in Haregeweyn et al.^[^ ^11^ ^]^

For the second dataset, the state of dissemination of SLM practices was evaluated by using information extracted from the World Overview of Conservation Approaches and Technologies^[^ ^26^ ^]^ database (https://explorer.wocat.net/), which produced a dataset of 1513 SLM practices. The WOCAT SLM database is the primary recommended database by the UNCCD for the reporting of SLM best practices.^[^ ^27^ ^]^

The third dataset comprised a list of 981 available SLM‐related policy and legal instruments. In the present context, “policy and legal instruments” refers to matters of soil governance, encompassing national and local government policies, strategies, and decision‐making processes regarding soil utilization. The list of legal instruments, each of which was undergone validation by national experts to ensure their relevance and currency, were sourced from the FAOLEX Database and the EU Soil Wiki via the FAO Soils Portal (http://www.fao.org/soils‐portal/soilex/soil‐keywords/soil‐conservation/en/).

**Figure 1:**
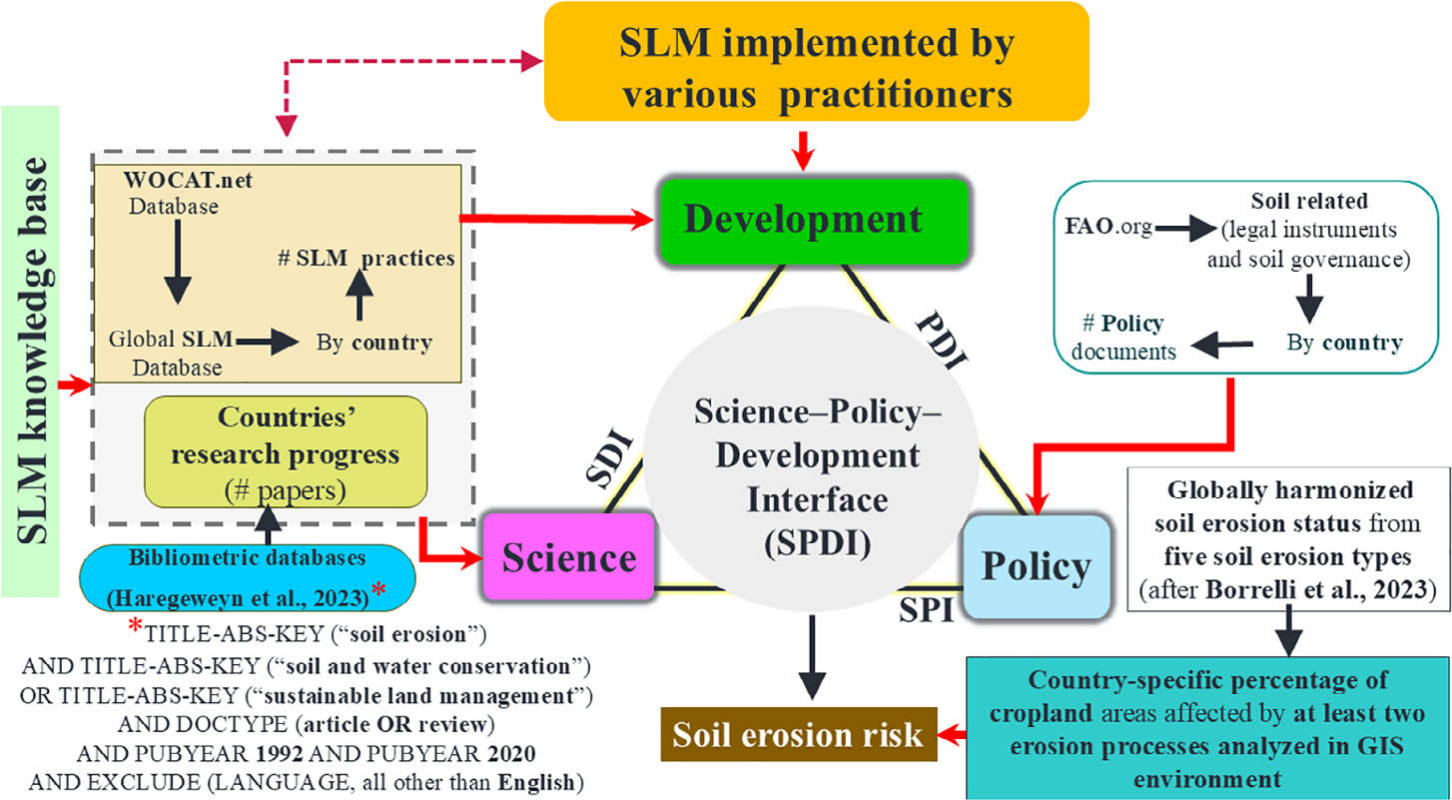
The multicriteria framework used in the present study to evaluate the science–policy (P)–development (D) interface (I) with respect to managing the soil‐erosion risk of countries. The framework integrates coevolving sustainable land management (SLM) research, land governance, and development practice from a multi‐stakeholder perspective, as well as scientific databases (e.g., Web of Science and Scopus), technological databases (e.g., World Overview of Conservation Approaches and Technologies database), and effective policy documents (FAO Soil Conservation Legal Instruments), for wider SLM implementation by various practitioners.

The fourth and final dataset was the current country‐level risk of soil erosion, which was compiled by re‐analysis of the gridded global soil‐erosion map originally presented by Borrelli et al.^[^ ^28^ ^]^ By using these four datasets, a multicriteria framework that incorporates various indices (**Figure** **1**) was constructed for evaluation of the sustainability of current SLM practices. More details are given in the following sections.

### 2.2 Assessment of the Linkages of SLM Dissemination, Science, and Policy with Soil‐Erosion Risk

The sustainability of current SLM practices was assessed by using a multicriteria framework (Figure 1) with respect to the following four relationships:

- The science–development (SD) linkage, which embeds research centrally within the development praxis for effective upscaling;
- The SP linkage, which promotes dialogue between scientists and policymakers by centrally embedding research within policy practice;
- The policy–development (PD) linkage, which allows SLM initiatives by communities and other stakeholders to be visible to policymakers and creates opportunities for private‐sector investment;
- The science–development–policy interface, which establishes a shared platform encompassing the three interface pairs, fostering enhanced relationships among stakeholders for centralized coordination in co‐creation of knowledge, managing and disseminating SLM knowledge and data. In addition, this interface facilitates capacity development to seamlessly integrate insights on global development and the science of SLM. Description of how these linkages were assessed is given in the following section.

### 2.3 Methodology for Assessing Erosion Risk and Indices of SLM Dissemination, Science, and Policy

Indices with summary statistics in ArcGIS Pro environment^[^ ^29^ ^]^ were developed and mapped to clarify the degree of SPDI and the opportunities and challenges on SLM arising from the linkages of the three interface pairs, science–policy interface (SPI), science–development interface (SDI), and policy–development interface (PDI) were analyzed. Based on the corresponding indices, solutions that can narrow the observed gaps for tackling future SLM challenges were proposed.

Globally harmonized erosion risk data were employed from Borrelli et al.,^[^ ^28^ ^]^ which included the combined erosion risk of five erosion types tailored specifically to cropland, and these data were used as a proxy for assessing erosion risk at the country level. The five soil erosion types were water erosion due to inter‐rill and rill, gully erosion, tillage erosion, wind erosion, and erosion due to root crop harvesting. Borrelli et al.^[^ ^28^ ^]^ used a multimodel approach to estimate the spatial distribution of cropland susceptibility to these erosion types, categorized into five classes: very low, relatively low, moderate, relatively high, and very high. Given the concurrent moderate to higher susceptibility across all erosion processes, the country‐specific percentage of cropland areas affected by at least two erosion processes were determined. These percentages were then classified as low, moderate, or high risk by using the variance minimization classification scheme reported by Jenks and Caspall.^[^ ^30^ ^]^ This classification scheme was selected as it works iteratively to group data values in a way that minimizes within‐class variance and maximizes between‐class variance.

Country‐specific total number of SLM‐related journal articles, soil conservation‐related legal instruments, and SLM approaches and technologies were used as indices for constructing index maps associated with science, policy, and development for specific countries. Initially, the three indices were categorized into three classes—low, moderate, or high—by using the variance minimization classification scheme.^[^ ^30^ ^]^ Then, the PDI, SDI, and SPI were formulated by combining pairs of index maps. If the two index maps shared the same class (e.g., low for policy and low for development), the combined index (i.e., PDI) was assigned that common class (low). If the two index maps differed by one order (e.g., moderate for policy and high for development), the lower class (moderate) was assigned to the combined index. If the two index maps differed by two orders (e.g., low for policy and high for development), the combined index was assigned the moderate class. The SPDI was examined by combining all three index maps into a single map. If the three index maps exhibited the same class (e.g., low, low, and low), that class determined the class that was assigned (in this case, low would be assigned). If two of the three index maps exhibited the same class (e.g., low and low) and the third index differed by one order (e.g., moderate), the two index maps with the same class determined the assigned class (e.g., low). If two of the three index maps exhibited the same class (e.g., low and low) and the third index differed by two orders (e.g., high), or if the three index maps had different classes (e.g., low, moderate, or high), the moderate class was assigned.

### 2.4 Framework to Enhance the Science–Policy–Development Linkages in SLM

Transdisciplinary research, which was characterized by process phases, knowledge types, and the intensity of involvement of practitioners, is a key component of sustainability science (Anzai et al., 2012).^[^ ^31^, ^32^, ^33^, ^34^, ^35^ ^]^ Like many other sectors, SLM research, development, and policy formulation often adopt their own processes with limited integration between one another. Thus, a framework to integrate these domains was needed. In research and development projects of SLM, the concept of transdisciplinary approach should be seen beyond the three components to include other aspects of the research project such as development of working documents, research communication, and ex‐post evaluation. Thus, in this study, to narrow the potential SPDI gap, a project cycle management‐based transdisciplinary research–development approach was proposed (EC, 2001).^[^ ^36^, ^37^ ^]^

The proposed framework uses a project cycle that comprises the following seven sequential stages: 1) shared research framing, 2) research grant acquisition, 3) co‐creation of integrated and transferable practices, 4) harmonization of tools and metadata, 5) development of policy instruments, 6) ensuring research communication, and 7) ex‐post evaluation. All relevant constructions were included by consulting expert opinions, literature reviews, and conducting case studies. Active engagement of researchers, practitioners, and policymakers at all project stages—problem identification, data collection, analysis, implementation, and evaluation—is essential to align scientific knowledge with real‐world applications.^[^ ^32^ ^]^ For instance, the framework benefited from multiple stakeholders’ workshops was held in November 2022 and March 2023 as part of the Science and Technology Research Partnership for Sustainable Development project (SATREPS), international joint research targeting global issues such as desertification and climate change. The workshops were attended by stakeholders from Japanese and Ethiopian research institutes, the Ethiopian Ministry of Agriculture, and international development partners such as German Development Cooperation and Japan International Cooperation Agency. As part of the standardization process, operational definitions of all key components were provided to ensure that they are universally understood and applied (see details in Section 3.2).

The reliability of the framework was discussed in the context of selected countries, complemented with published results, and expert opinions, including those of experienced co‐authors with firsthand knowledge of their respective regions. Moreover, feedback was incorporated by presenting the framework at various local and international conferences, such as the 2022 American Geophysical Union Meeting,^[^ ^38^ ^]^ the International Workshop on Mollisols Erosion, and Degradation held in China,^[^ ^39^ ^]^ and the UNCCD COP‐16 held in Saudi Arabia.^[^ ^40^ ^]^ Likewise, in a more local context such as Ethiopia, feedback was obtained from stakeholders and experts to confirm the framework's relevance and clarity through research workshops conducted as part of the SATREPS collaborative research project, which is discussed under Section 4.2. Reed et al.^[^ ^41^ ^]^ stressed the use of such mixed methods enhances data reliability while triangulation techniques validate results across different disciplines.

## 3 Results

### 3.1 Country‐Level Correspondences between Soil Erosion Risk, and Science, Policy, and Development Interfaces

#### 3.1.1 Current Status Regarding SLM‐Related Scientific Publications

**Figure 2:**
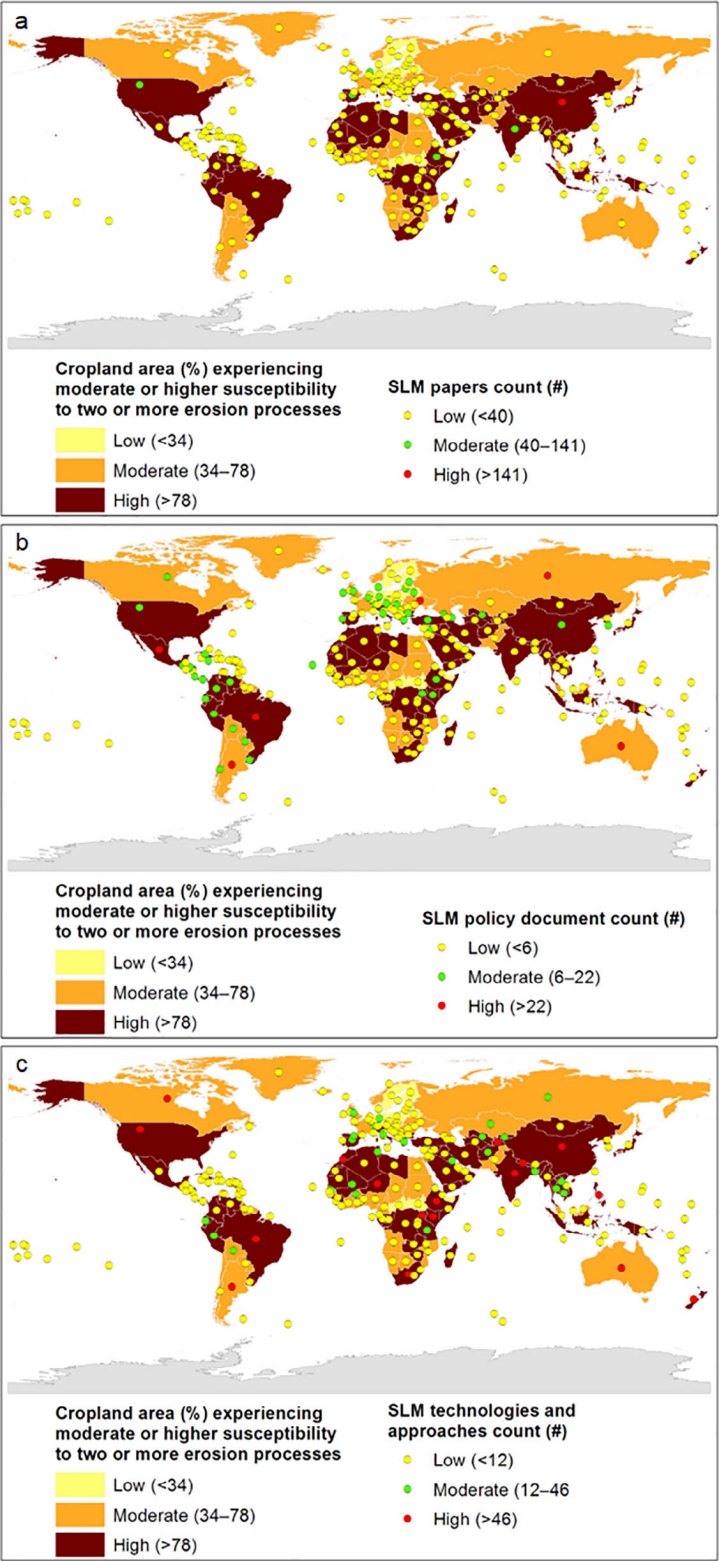
a–c) Correspondence between soil erosion risk and the indicated sustainable land management number of research publications, policy documents, and technologies and approaches at the country level.

**Figure** **2** shows global maps overlaid with keys showing the percentage of cropland area currently experiencing low, moderate, or high susceptibility to two or more erosion processes, and the numbers of SLM‐related research papers, policy documents, and technologies and approaches produced by 236 countries. A total of 46 (20%), 60 (25%), and 130 (55%) countries currently have a low, a moderate, and a high erosion risk, respectively (**Table** **1**).

**Table 1:** Summary statistics for the correspondence between soil erosion risk and the number of sustainable land management (SLM)‐related research papers, policy documents, and technologies and approaches at the country level (values are the number of countries).

a. Soil erosion risk vs SLM research					
		SLM research publications			
		Low	Moderate	High	Total
Soil erosion risk	Low	45	1	0	46
	Moderate	59	1	0	60
	High	126	3	1^a)^	130
	Total	230	5	1	236
b. Soil erosion risk vs SLM policy					
		SLM policies			
		Low	Moderate	High	Total
Soil erosion risk	Low	39	7	0	46
	Moderate	47	9	4	60
	High	102	26	2^b)^	130
	Total	188	42	6	236
c. Soil erosion risk vs SLM dissemination/development					
		SLM technologies and approaches			
		Low	Moderate	High	Total
Soil erosion risk	Low	45	1	0	46
	Moderate	49	8	3	60
	High	100	16	14^c)^	130
	Total	194	25	17	236

^a)^ China;

^b)^ Mexico and Brazil;

^c)^ China, Brazil, Ethiopia, India, Kenya, Morocco, Nepal, Niger, New Zealand, Philippines, South Africa, Tajikistan, Uganda, and USA.

When the numbers of countries with low, moderate, and high risks of soil erosion were tabulated against the number of scientific publications related to SLM, the erosion risk aligned with the number of scientific publications for 47 countries (20%) (Table 1a). Only one country (China) showed a high erosion risk and a high number (i.e., 473) of scientific publications related to SLM. A total of 188 countries (80%) showed a moderate or high risk of erosion but only a low or moderate number of scientific publications related to SLM.

When soil erosion was tabulated against the number of SLM‐related policy documents, 50 countries (21%) showed the same level of erosion risk and number of SLM‐related policy documents (Table 1b). Mexico and Brazil were the only countries that showed a high erosion risk and a matching high policy level. Despite 11 countries (5%) showing a higher policy level than erosion risk level, 175 countries (74%) showed a moderate or high erosion risk that was not matched with their policy level.

When soil erosion was tabulated against the number of SLM‐related technologies and approaches, 67 countries (28%) showed the same level of erosion risk and number of SLM‐related technologies and approaches (Table 1c). A total of 14 countries (6%; China, Brazil, Ethiopia, India, Kenya, Morocco, Nepal, Niger, New Zealand, Philippines, South Africa, Tajikistan, Uganda, and USA) showed a high erosion risk and a high number of SLM‐related technologies and approaches. Despite 4 countries (2%) showing an SLM technology and approach level that was higher than their erosion risk, 165 countries (70%) showed an SLM technology and approach level that was lower than their erosion risk.

These findings highlight the fact that considerable gaps exist between erosion risk and the number of SLM‐related scientific publications, policies, and technologies and approaches that are produced at the country level. Understanding the inter‐relationships among these three aspects, which are discussed in the subsequent sections, is expected to give insights into how integrated soil erosion mitigation strategies can be designed and implemented in these countries.

#### 3.1.2 Soil Erosion Risk and SPI

**Figure 3:**
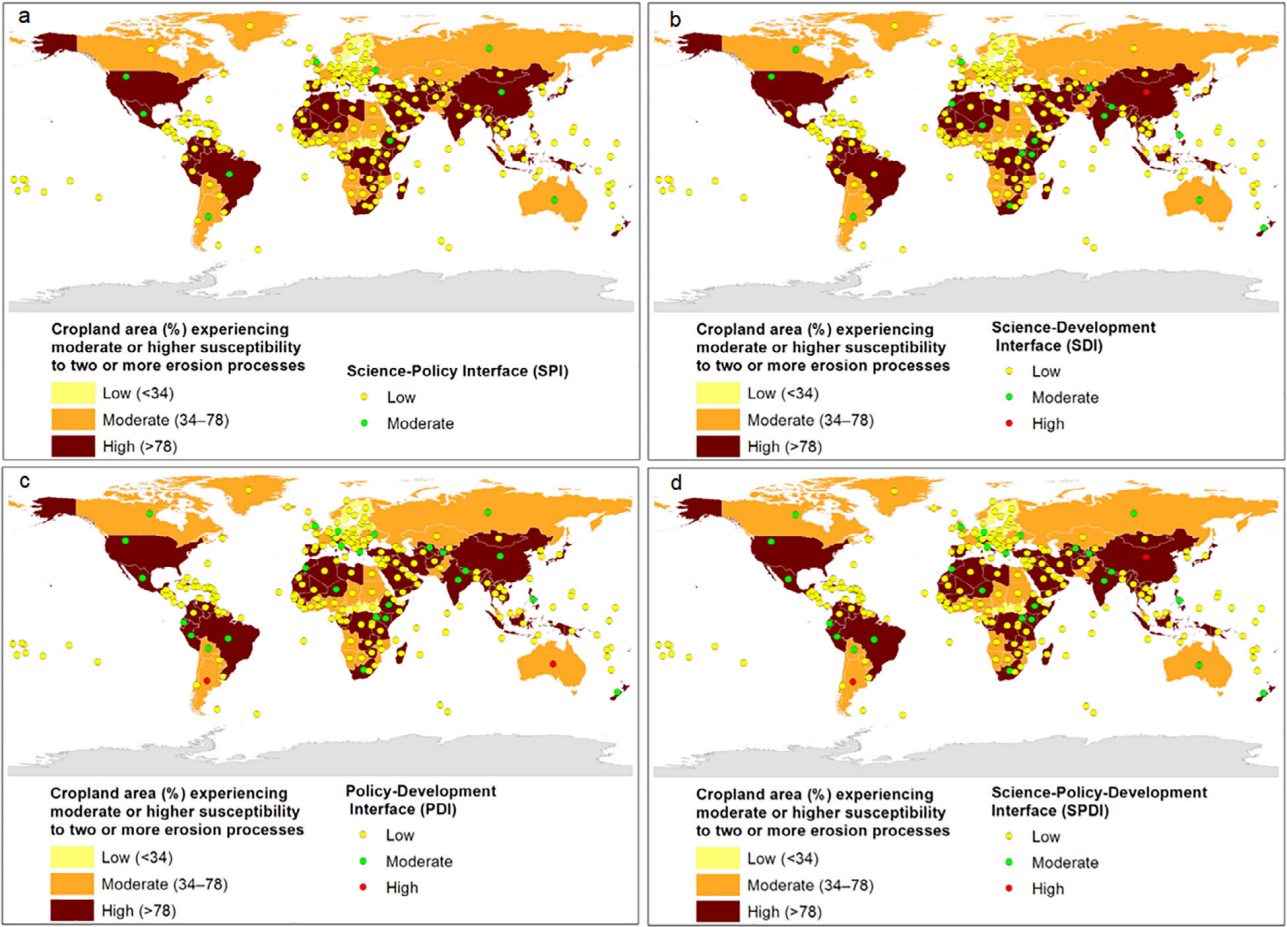
a–d) Correspondence between soil erosion risk and the indicated science, policy, and development interfaces at the country level.

**Figure** **3**a and **Table** **2**a show the correspondence between soil erosion risk and SPI at the country level. A total of 226 countries (96%) showed a low SPI level, and 10 countries (4%) showed a moderate SPI level; no countries showed a high SPI level. For 51 countries (22%), their SPI level currently matches their erosion risk. No country had an SPI level better than its level of erosion risk, whereas 185 countries (78%) are facing a moderate‐to‐high erosion risk that is not matched by their SPI. Of particular concern are the 125 countries (53%) experiencing a high erosion risk but possessing a low SPI (see country lists in Table S1 in the Supporting Information).

**Table 2:** Summary statistics for the correspondence between soil erosion risk and science–policy interface (SPI), science–development interface (SDI), policy–development interface (PDI), and science–policy–development interface (SPDI) at the country level (values are the number of countries).

a. Soil erosion risk vs SPI by country					
		SPI			
		Low	Moderate	High	Total
Soil erosion risk	Low	46	0	0	46
	Moderate	55	5	0	60
	High	125	5	0	130
	Total	226	10	0	236
b. Soil erosion risk vs SDI by country					
		SDI			
		Low	Moderate	High	Total
Soil erosion risk	Low	46	0	0	46
	Moderate	56	4	0	60
	High	117	12	1^a)^	130
	Total	219	16	1	236
c. Soil erosion risk vs PDI by country					
		PDI			
		Low	Moderate	High	Total
Soil erosion risk	Low	45	1	0	46
	Moderate	53	5	2	60
	High	109	21	0	130
	Total	207	27	2	236
d. Soil erosion risk vs SPDI by country					
		SPDI			
		Low	Moderate	High	Total
Soil erosion risk	Low	45	1	0	46
	Moderate	53	6	1	60
	High	109	20	1^a)^	130
	Total	207	27	2	236

^a)^ China.

#### 3.1.3 Soil Erosion Risk and SDI

Figure 3b and Table 2b show the correspondence between soil erosion risk and SDI at the country level. A total of 219 (93%), 16 (7%), and 1 (0.4%; China) countries showed a low, a moderate, and a high SDI level, respectively. While 51 countries (22%) have SDI levels that are aligned with their erosion risk, notable discrepancies exist in many regions (see also Table S1 in the Supporting Information). No country showed an SDI level that was higher than their erosion risk, and 185 countries (78%) had a moderate or high erosion risk level that was not consistent with their SDI level. Only one country (China) showed a high level of erosion risk and had a corresponding high SDI level.

#### 3.1.4 Soil Erosion Risk and PDI

Figure 3c and Table 2c show the correspondence between soil erosion risk and PDI at the country level. A total of 207 (88%), 27 (11%), and 2 (0.9%; Argentina and Australia) countries showed a low, a moderate, and a high PDI level, respectively. Fifty countries (21%) had a PDI level that matched their level of erosion risk. Despite 3 countries (1%) showing a PDI level that was higher than their erosion risk, 183 countries (78%) had a PDI level that was below their level of erosion risk.

#### 3.1.5 Soil Erosion Risk and SPDI

Figure 3d and Table 2d show the correspondence between soil erosion risk and SPDI at the country level. A total of 207 (88%), 27 (11%), and 2 (0.9%) countries showed a low, a moderate, and a high SPDI, respectively. Fifty‐two countries (22%) had a SPDI level that matched their erosion risk. Despite 2 countries (0.9%) showing an SPDI level that was better than their erosion risk, 182 countries (77%) showed an SPDI level was worse than their erosion risk level. Only one country (0.4%; China) showed a high erosion risk level and a corresponding high SPDI level. In addition, only one country (0.4%; Argentina) showed a high SPDI level that was greater than their moderate erosion risk level, signaling that this country has better science–policy–development linkages than the others (see discussion in Section 4.1).

#### 3.1.6 SPDI and the Individual Interfaces

**Table** **3** shows the correspondence between the SPDI level and PDI, SDI, and SPI levels. SPDI was aligned with PDI in 234 countries (99%), with SDI in 223 countries (95%), and with SPI in 215 countries (91%), implying the greater need for science‐based policy and development to mitigate the risk of erosion‐based land degradation. In this regard, particular attention should be given to the 109 countries (46%) identified here that have a high erosion risk but a low SPDI level (Table 2d; Table S1, Supporting Information).

**Table 3:** Summary statistics for the correspondence between science–policy–development interface (SPDI) and science–development interface (SDI), policy–development interface (PDI), and science–policy interface (SPI) at the country level (values are the number of countries).

a. PDI vs SPDI					
		SPDI			
		Low	Moderate	High	Total
PDI	Low	207	0	0	207
	Moderate	0	26	1	27
	High	0	1	1^a)^	2
	Total	207	27	2	236
b. SDI vs SPDI					
		SPDI			
		Low	Moderate	High	Total
SDI	Low	207	12	0	219
	Moderate	0	15	1	16
	High	0	0	1^b)^	1
	Total	207	27	2	236
c. SPI vs SPDI					
		SPDI			
		Low	Moderate	High	Total
SPI	Low	207	19	0	226
	Moderate	0	8	2	10
	High	0	0	0	0
	Total	207	27	2	236

^a)^ Argentina;

^b)^ China.

### 3.2 Transforming Current SLM Initiatives into a Transdisciplinary Framework

**Figure 4:**
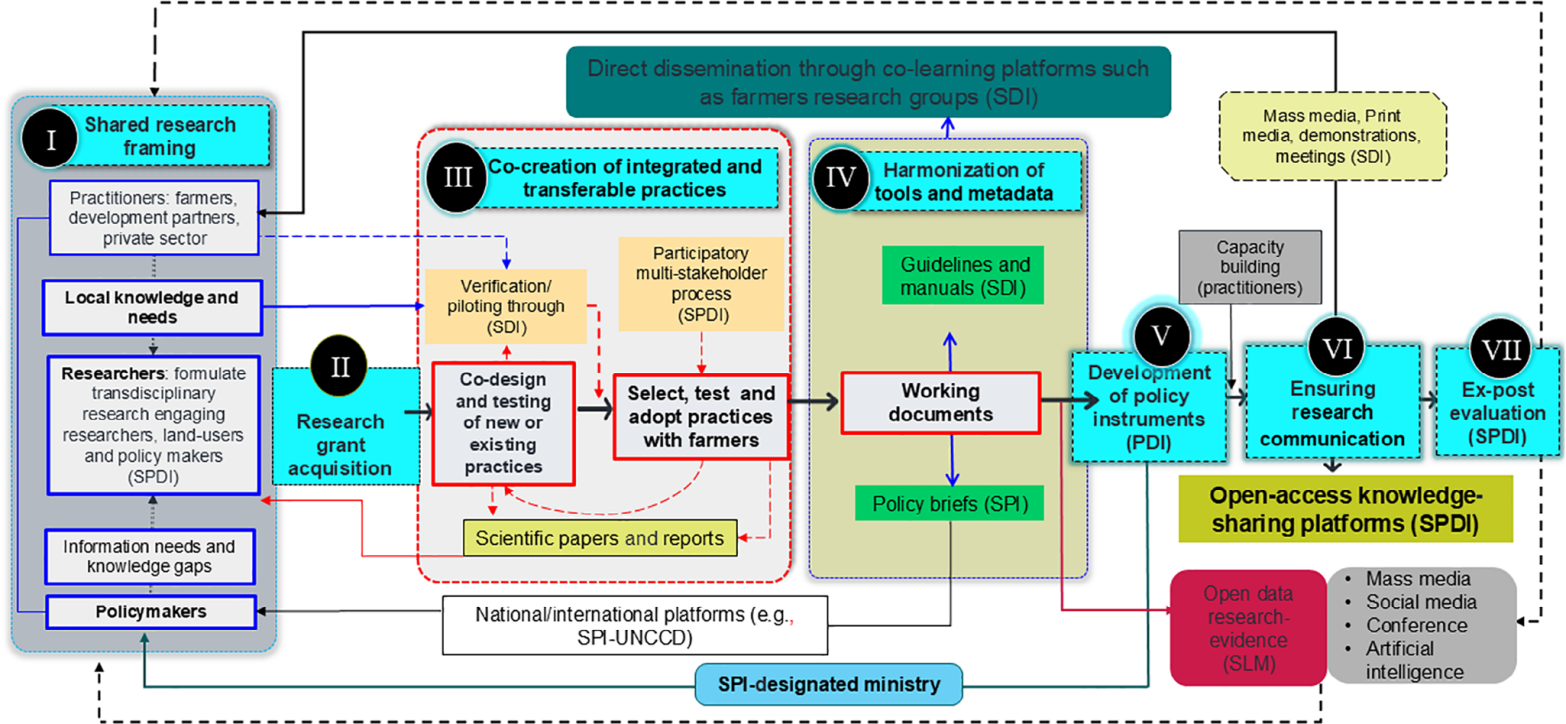
Our proposed transdisciplinary framework that bridges the gap between science, policy, and development by integrating co‐evolving local and scientific knowledge, as well as incorporating multiple stakeholder perspectives in the development and dissemination of sustainable land management (SLM) practices. SPDI; science–policy–development interface.

As highlighted in Section 3.1.6, the low SPDI with respect to SLM arises from a weak PDI, followed by SDI and SPI. This suggests the need for a transdisciplinary framework to guide the development of future research and development projects, including their funding and timelines. Transdisciplinary research promotes collaboration between researchers and practitioners, uniting scientific and social expertise to tackle environmental challenges (Anzai et al., 2012).^[^ ^32^, ^42^, ^43^ ^]^ The need for clear consensus on what transdisciplinarity is or how its quality can be evaluated has been emphasized in Wickson et al. (2006),^[^ ^44^ ^]^ being characterized with its complex and multidimensional problems, shared and evolving methodology that has fused different disciplinary approaches. While many transdisciplinary projects follow context‐specific approaches, Brandt et al.^[^ ^31^ ^]^ suggest that it may be valuable to develop a common terminology, focused communication platform, and commonly shared research frameworks. Therefore, we constructed a project cycle management‐based transdisciplinary research and development framework to bridge science, policy, and development. The framework covers the seven stages (**Figure** **4**), each of which is discussed in the following sections.

#### 3.2.1 Shared Research Framing

The interest in generating research‐based evidence for the efficacy of SLM technologies is growing,^[^ ^22^ ^]^ implying that improving the linkages between science and both policy and development should be a priority. Many policymakers argue that scientific research should align more with societal needs for impactful policy outcomes. However, policymakers often seek quick solutions, underestimating data collection and development time for conducting research. This underestimation creates “invisible boundary lines” around research, influencing content, approach, and timing.^[^ ^45^ ^]^ Policymakers are often unaware of community‐initiated SLM projects, lacking institutional support for wider adoption.^[13]^ Bridging the scientist–practitioner–‐policymaker gap in SLM requires a middle ground that can accommodate both urgent solutions and long‐term concerns, as well as equitable navigation of power relations among actors^[^ ^6^, ^46^ ^]^ describes a few collaborative pathways for iterative engagement in reflexive dialogues to foster the development of shared ideas and actions beyond initial possibilities. This fostering is accomplished by elevating marginalized agendas, actively navigating conflicting perspectives, and promoting mutual respect through diverse exploration and learning.

The first phase of our transdisciplinary research–development framework is the initial collaborative research phase to establish a shared framework that will guide the core research process. Activities include identifying real‐world problems, defining research objectives and questions, creating a knowledge framework, and forming a research and development team. Crucially, this phase turns the identified real‐world issues into researchable boundary objects that can be used to change society and be integrated into scientific knowledge (e.g., Clark et al., 2011).^[^ ^47^ ^]^ Team building is crucial at the start of transdisciplinary research projects to ensure success and synthesize evidence of what works, where, and for whom.^[^ ^30^, ^48^, ^49^ ^]^ Fam et al.^[^ ^50^ ^]^ explored divergent expectations and tensions, including “I” versus “we”, disciplinary versus transdisciplinary, and research versus learning, all stemming from individual and team differences, and emphasized the need for effective teamwork and communication to address these divergences.

#### 3.2.2 Research Grant Acquisition

Research funding significantly shapes the landscape of scientific inquiry in terms of topic, geographic focus, participant eligibility, and evaluation criteria and has a profound impact on project outcomes.^[^ ^33^, ^51^ ^]^ However, transdisciplinary research faces formidable challenges within the existing funding paradigms. Funding schemes often favor short‐term, small‐scale projects and are often misaligned with the extended timelines and resource requirements of transdisciplinary endeavors.^[^ ^52^, ^53^ ^]^ Moreover, the preference toward individual researchers and single‐discipline projects sidelines the collaborative nature of transdisciplinary research, hampering its development.^[^ ^54^, ^55^, ^56^ ^]^ Funding allocation also tends to prioritize scientific curiosity over addressing broader societal issues, hindering the application of transdisciplinary solutions.^[^ ^57^ ^]^ Meanwhile, the geographical concentration of research funding in specific countries hampers progress in developing nations, making external collaborations crucial.^[^ ^11^, ^58^ ^]^ Addressing these challenges requires structural changes, larger‐scale funding models involving multiple institutions, industry collaboration,^[^ ^45^ ^]^ and political and public support to nurture key researchers with broad perspectives (Anzai et al., 2012). In practice, transdisciplinary projects frequently piece together funding from various sources, which may be contributed by different partners, including researchers and practitioners. The emergence of large‐scale research funding initiatives such as Future Earth and the Horizon Europe highlights the important role research plays in generating transformative knowledge, emphasizing transdisciplinary research (Lawrence, 2015).^[^ ^59^ ^]^ For instance, the EU has adopted an ambitious funding framework for research and innovation out of which “A Soil Deal for Europe” is one aiming to achieve the EU's policy objective to have all soils healthy by 2050 with expected funding worth 1 billion Euro by 2030.^[^ ^60^ ^]^

#### 3.2.3 Co‐Creation of Integrated and Transferable Practices

The third stage in our framework is the co‐production of both research and new practices. In this phase, a set of integrative methods is adopted, further developed, and applied to facilitate the differentiation and integration of the different bodies of knowledge coming together in the process. This stage involves the following main activities, co‐design, and testing of new or existing practices, piloting of research results, and multi‐stakeholder analysis, each of which are discussed in detail in the following sections.

*Co‐Design and Testing of New or Existing Practices*: The findings from our assessment of erosion risk versus research efforts underscore the urgent need for multiple evidence‐based policies and development strategies to address the risks associated with land degradation due to soil erosion. A major challenge for researchers at this stage will be working with multiple forms of knowledge and practice (science as well as indigenous, local, and practitioner‐based knowledge, among others) to develop “user‐inspired” and “user‐useful” management approaches.^[^ ^43^, ^61^ ^]^ This requires designing suitable mechanisms for local adaptation and service delivery, along with research approaches that facilitate co‐learning among the participants from research, development, local communities, nonprofit and private sectors.^[^ ^62^ ^]^ The landscape of applied research has witnessed a significant evolution where the traditional linear research model, characterized by technology development by researchers and its dissemination through extension agents but also by its contribution to agricultural productivity stagnation^[^ ^63^ ^]^ has been replaced by the “research‐for‐development” or “research‐for‐transformation” principles within the innovation systems framework, which have facilitated increased technology adoption.^[^ ^64^ ^]^ However, the “research‐for‐development” approach, which assumes that science inherently leads to progress, may encounter challenges associated with limited demand within the development community.^[^ ^65^ ^]^ Therefore, it is essential to view the “research‐for‐development” approach not only as “change through knowledge” but also as “knowledge through change”,^[^ ^61^, ^66^, ^67^ ^]^ which has led to the development of the concept of “research‐in‐development.” This approach underscores the integration of local wisdom and scientific expertise, promoting adaptive, and context‐sensitive land management practices^[^ ^55^, ^68^, ^69^ ^]^ and fostering a shift toward a demand‐driven paradigm that takes into account market dynamics, social capital, institutional factors, and innovation capacity (Maru et al., 2018).^[^ ^70^ ^]^ Furthermore, the co‐design process encourages continuous learning, iterative feedback loops, and the consideration of social and ecological variables, making it a dynamic and responsive approach to land management that can enhance sustainability and resilience.^[^ ^71^ ^]^ The co‐production of knowledge is best embraced within a transdisciplinary research framework.^[^ ^8^, ^16^, ^72^ ^]^ To be effective, research and development programs require insights into options, delivery mechanisms, and enabling environments, necessitating systematic research and resource allocation for co‐design and knowledge generation.

*Verification/Piloting of Transferable Practices*: Piloting research results and practices refers here to the process of testing and validating the findings of a research study before implementing them on a larger scale. The main focus of research has been on the generation of technology rather than on integration, packaging, and upscaling within a holistic interdisciplinary framework.^[^ ^66^ ^]^ In addition, the linkage between research and extension institutions is lacking, particularly in the transfer of feedback from farmers to researchers.^[^ ^73^, ^74^ ^]^ A review by Landini et al.^[^ ^75^ ^]^ identified complex interrelated issues within the linkage between agricultural research, rural extension, and farmers that impact institutional structures, incentive systems, and innovation concepts and influence stakeholder interactions. The linkage problem is particularly pressing for SLM technologies with high time and resource demands under poor endowments and diverse ecological conditions.^[^ ^11^ ^]^ Several approaches have been adopted to integrate agricultural research and extension systems. For instance, on‐farm research has been used to foster collaboration among stakeholders for technology verification within farming systems^[^ ^76^ ^]^ Such research serves as a meeting point where fine‐tuned technologies can be disseminated widely by extension agencies (Paudel, 2013).^[^ ^77^ ^]^ Hauser et al. (2015)^[^ ^78^ ^]^ have highlighted the importance of collaborative research involving a multidisciplinary research team, extension workers, and groups of farmers to jointly conduct research on farmers’ fields on selected topics based on farmers’ needs. Such collaborative research allows researchers to refine their approach, address challenges, and maximize the chances of successful upscaling. Farmer research and extension groups also help to efficiently develop, adapt, and disseminate technologies across varied agroecological zones, a task too resource‐intensive for researchers alone.^[^ ^79^, ^80^ ^]^

*Participatory Multi‐Stakeholder Analysis to Select and Adopt Practices with Land Users*: An essential element in co‐creating integrated and transferable practices is a participatory, multi‐stakeholder process with land users to select, test, and adopt practices. Schwilch et al.^[^ ^81^ ^]^ proposed a structured, resource‐efficient, multi‐stakeholder learning process for SLM. It involves stakeholder workshops to identify potential solutions, followed by documentation, sharing, and evaluation using a standardized questionnaire. The most promising options are then chosen for local implementation, fostering consensus, social learning, and wider SLM practice adoption.^[^ ^82^ ^]^ There can be challenges that need consideration for the full realization of multi‐stakeholder process within transdisciplinary research such as conflicts between diverse disciplines, resource intensiveness, complex power dynamics, and the lack of standardized evaluation criteria.^[^ ^30^, ^31^, ^83^ ^]^

#### 3.2.4 Harmonization of Tools and Metadata

In land management, the harmonization of tools and data refers to standardizing data and methodologies to ensure consistent and compatible collection, processing, and analysis of land‐related information^[^ ^40^, ^84^ ^]^ stresses the need to balance real‐world complexity with clear communication in both local and international languages. Nowak and Rosenstock^[^ ^72^ ^]^ stressed the need to tackle existing imbalances between researchers, policymakers, and development actors to create a proper communication platform and medium and whereby joint development of tools, guidelines, harmonized SLM practices, and a metadata‐sharing platform is widely accessible.

*Provision of Appropriate Tools Such as Guidelines or Manuals*: The lack of evidence‐based guidelines or manuals has been a significant barrier to translating research results into effective practice.^[^ ^85^ ^]^ Without clear and well‐documented guidelines, the dissemination of research findings and their practical application can be challenging. To be effective, these manuals must provide land users with specific, straightforward, and practical details about SLM technologies and approaches.^[^^]^ A common challenge, however, is that such manuals are often authored primarily by experts or consultants, without substantial input from researchers and key stakeholders who possess valuable on‐the‐ground experience and insights, which can result in a lack of consideration for local agroecology and specific land‐use contexts.^[^ ^86^ ^]^ Mann and Schafer^[^ ^87^ ^]^ have stressed the importance of transdisciplinary research processes to intensify the interactions among stakeholders, and the need for appropriate manual development to influence acceptance and adoption of generated research products.

#### 3.2.5 Development of Policy Instruments

The fifth stage in our framework is the development of policy instruments. The analysis presented in Section 3.1 highlights a lack of evidence‐based policies to tackle erosion risk in many countries. This aligns with Carter's^[^ ^45^ ^]^ observation that translating knowledge into practical political measures is challenging. Prager et al.^[^ ^88^ ^]^ stress the importance of engaging the scientific community in co‐creating solutions with policymakers to inform national decisions. They advocate for evidence‐based land resource management, local innovation, and validation before scaling up. Dwyer et al.^[^ ^89^ ^]^ underscore the complementary roles of policy, private actors, and communities in promoting beneficial outcomes, strengthening ecosystem services, and ensuring sustainability.

The UNCCD SPI report^[^ ^2^ ^]^ emphasizes the need for an enabling environment at national and subnational levels to overcome barriers to large‐scale SLM implementation. This involves integrating SLM into policy, planning, and finance decisions, supporting economic incentives, improving land tenure security, and fostering stakeholder engagement.^[^ ^90^ ^]^ Zdruli et al.^[^ ^91^ ^]^ argue that successful mitigation of land degradation and desertification requires the right policy instruments that enable an integrated ecosystem management strategy. Despite the significance of land degradation, this field has received less support from the private sector and funding compared to biodiversity and climate change. To support land management practices with broader benefits, Baumber et al.^[^ ^92^ ^]^ suggest aligning land degradation neutrality goals with existing market‐based instruments for carbon, biodiversity, and bioenergy. The UNCCD Strategic Framework 2018–2030 offers an opportunity to draw lessons from existing environmental markets^[^ ^1^ ^]^ and advance efforts toward land degradation neutrality. Carter^[^ ^45^ ^]^ highlighted the challenge of effectively translating research to national and international bodies like the High‐Level Political Forum on Sustainable Development, underscoring the need to creating enabling policies.

#### 3.2.6 Ensure Research Communication

The sixth stage in our framework is ensuring that research communication links scientific knowledge with practical implementation that includes methods of communication, improved access to data or information.

*Knowledge Communication*: Kennett^[^ ^36^ ^]^ stresses that the value of research is lost if it is not communicated to the relevant stakeholders. Oladele and Sakagami (2004)^[^ ^93^ ^]^ reviewed research‐extension linkages in various countries, highlighting strengths such as strong collaboration and extensive coverage, and weaknesses like limited alternative methods and bureaucratic issues. Ho (1996)^[^ ^94^ ^]^ advocates for a participatory approach to agricultural extension involving farmers as active experimenters, fostering sustainable learning when combined with farmer‐to‐farmer extension, often taught as a low cost and effective extension approach. Strengthening research‐extension connections is essential, especially in resource‐poor regions, where technology needs are not demand‐driven and farmer requirements are expressed through government policy.^[^ ^80^ ^]^

Furthermore, deploying integrated use of mass media, conferences, social media, and artificial intelligence (AI) can help facilitate knowledge exchange across multiple stakeholders in quick and cheap ways. Mass media have long played a key role in raising awareness of global challenges, making research accessible to both policymakers and the public.^[^ ^95^, ^96^ ^]^ Similarly, conferences serve as essential platforms for dialogue, fostering collaboration between researchers and practitioners to critique findings and develop innovative solutions. Cairney^[^ ^97^ ^]^ emphasizes the need for persuasive strategies, coalitions, and storytelling to engage policymakers effectively in evidence‐based decision‐making. In recent years, social media platforms like Twitter, ResearchGate, and LinkedIn have accelerated research communication by enabling instant, transparent, and interactive knowledge sharing across diverse audiences (Waechter, 2022).^[^ ^98^, ^99^ ^]^ Additionally, AI tools like ChatGPT enhance research dissemination by generating multilingual summaries, abstracts, and social media posts, broadening accessibility.^[^ ^100^ ^]^ However, users must exercise caution, ensuring human oversight for accuracy and clarity.^[^ ^101^ ^]^ By integrating these tools, transdisciplinary research can reach wider audiences, foster collaboration, and drive impactful solutions.

*Promoting Evidence‐Based, Open‐Access Data Sharing Platforms*: The International Science Council (ISC)^[^ ^102^ ^]^ strongly supports open access to research publications, data, and code to promote transparency in advancing sustainable development goals. To address soil erosion, similar initiative like that of the International Research Consortium (IRC; https://irc‐orcasa.eu/) for carbon sequestration is needed. The IRC, with its partners and regional nodes, uses an online platform to collect and share knowledge on soil carbon, providing better access to research, methods, and practices. Targeting researchers in low‐ and middle‐income countries, Research4Life (https://www.research4life.org/), a global access initiative offering free or low‐cost access to scientific literature has been initiated. This initiative can help bridge the information gap between developed and developing regions, fostering international cooperation and knowledge exchange in land management.^[^ ^103^ ^]^

#### 3.2.7 Ex‐Post Evaluation

The necessity, challenges, and key considerations for ex‐post evaluations of research projects are widely recognized in academic literature. Several studies highlight that monitoring, and evaluation must occur at each stage of the project cycle and throughout its lifespan to facilitate continuous improvements and enhance adaptability.^[^ ^32^, ^33^ ^]^ Among these, ex‐post evaluations—typically conducted within 3 years of project completion—are particularly critical yet often overlooked. These evaluations aim to assess project effectiveness and sustainability, extracting lessons and recommendations to improve ongoing and future initiatives^[^ ^58^ ^]^ while the eligibility and type of ex‐post evaluation often depend on the specific requirements of the funding agency.^[^ ^104^ ^]^

Despite their significance, ex‐post evaluations present unique challenges, particularly for transdisciplinary research. This type of research is highly context‐specific, time‐ and space‐dependent, and lacks a well‐defined peer community, resulting in the absence of standardized evaluation criteria—especially concerning the intended societal effects of transdisciplinary processes.^[^ ^105^ ^]^ Wickson et al.^[^ ^106^ ^]^ emphasize the importance of assessing how well transdisciplinary research incorporates key features such as problem focus, evolving methodology, and collaboration while addressing critical challenges, including integration, reflection, and paradoxes. Jahn et al.^[^ ^55^ ^]^ further stress the necessity of considering temporal, spatial, and systemic scales when evaluating research outcomes—distinguishing between short‐term versus long‐term (time), local versus global (space), and narrow versus broad systemic impacts (scope). For maintaining adaptability in addressing complex, evolving challenges, both retrospective strategic assessments—reflecting on past performance—and anticipatory assessments—designed to refine future transdisciplinary research—are essential.^[^ ^107^ ^]^ Additionally, evaluations must integrate both tactical dimensions, which focus on operational aspects such as collaboration effectiveness and knowledge integration, and strategic dimensions, which examine long‐term systemic changes and policy impacts.^[^ ^108^ ^]^

Measuring impacts on SLM projects presents unique challenges. These projects require long timeframes for environmental and social effects to emerge, complicating impact assessments (de Almeida et al., 2021).^[^ ^109^ ^]^ Additionally, evaluation criteria often vary across participants and knowledge systems, further increasing complexity.^[^ ^110^ ^]^ As a result, objective methodologies for assessing project productivity and interdisciplinary integration remain limited.^[^ ^105^, ^111^ ^]^ Traditionally, evaluations have relied on qualitative methods like peer review (Anzai et al., 2012), though many are narrow in scope, focusing on specific impacts or contexts (Almeida et al., 2021). Despite its qualitative nature, the most significant change (MSC) technique^[^ ^112^ ^]^ facilitates program evaluation by engaging stakeholders in storytelling‐based assessments, addressing questions such as, “Who did what?”, “When did the change occur?” and “What was the process?” Given the absence of a single optimal evaluation approach, Raymond et al.^[^ ^61^ ^]^ emphasize that evaluations should be systematic, reflexive, and cyclic, integrating multiple perspectives and methods to comprehensively address environmental management challenges.

## 4 Discussion

In this section, we present case studies from selected countries—China and Argentina—to understand how these countries could achieve better science–policy–development linkages concerning their respective soil erosion risks. Practical applications of the proposed transdisciplinary framework are discussed in the context of a successful interdisciplinary collaborative research and development project on SLM, which involved some of the authors of this paper, including the lead author. Lastly, we highlight some limitations that need to be considered when interpreting the findings.

### 4.1 SPDI and Soil Erosion Risk

Given the anticipated population growth and projected climate change by the end of this century, soil erosion by water is expected to increase by about 30–60%.^[^ ^19^, ^113^ ^]^ Thus, the 182 countries identified the present analysis with moderate‐to‐high erosion risk and low‐to‐moderate SPDI level (Table 2d) are in urgent need of better integration of science, policy, and development to deal with their soil erosion risk. In contrast, the experiences of China and Argentina are noteworthy and discussed here.

“China” in 1999, initiated the Grain‐for‐Green Program as a pilot program to address cropland set‐aside objectives aimed at augmenting forest cover and curbing soil erosion.^[^ ^114^ ^]^ The program was conducted in two phases from 1999 to 2010 and from 2014 to 2020. Widely recognized as one of the most ambitious conservation set‐aside endeavors, not only in China but globally,^[^ ^115^, ^116^ ^]^ the program has yielded significant results, with government data indicating that by 2013, the program had restored 27.8 million hectares of forest across 26 of China's 31 mainland provinces, substantially enhancing vegetation cover.^[^ ^117^ ^]^ Despite this progress, land‐use modification related problems leading to contradiction between land resource conservation and livelihood of local communities was reported, especially at the early state of the program.^[^ ^118^, ^119^ ^]^

“Argentin” has implemented several land‐management policies and practices focused on sustainable development and conservation. These include among others the Bonn Challenge to restore one million hectares by 2030 (IUCN, 2020),^[^ ^120^ ^]^ and the National Land Planning Policy, aimed at regulating land use to prevent degradation, and promote sustainable agriculture and forest conservation (SDSN, 2020;^[^ ^121^ ^]^ GIZ, 2021).^[^ ^122^ ^]^ Overall, Argentina has established a network of protected areas covering 22 million hectares, contributing to biodiversity conservation and sustainable tourism (Martinuzzi et al., 2018).^[^ ^123^ ^]^ Argentina also promotes sustainable agriculture practices like no‐till farming^[^ ^124^ ^]^ that could reduce soil erosion by water around 30–35%, which is the highest achievement worldwide.^[^ ^125^ ^]^ Furthermore, the government supports community‐based conservation initiatives and indigenous land rights to involve local communities in natural resource management and preserve biodiversity and cultural heritage.^[^ ^126^ ^]^ Finally, Argentina has implemented a specific national legislation to protect native forests (law 26331/07) by promoting their sustainable use and the development of more effective conservation strategies.^[^ ^127^ ^]^

### 4.2 Application of Our Proposed Transdisciplinary Framework to the SATREPS Project

While Argentina and China provide examples of relatively strong SPDI, many countries with the potential for strong SPDI continue to experience serious challenges with soil erosion and land degradation, for example, Ethiopia. In this section, we apply our proposed framework to a component of the SATREPS project that was conducted in Ethiopia (Table S2, Supporting Information).

Ethiopia is currently confronting a major challenge in relation to severe water erosion‐induced land degradation despite its potential for a high SPDI (Figure 3d; Table S1, Supporting Information). Several national SLM programs have been initiated, often with international donor support, primarily focusing on reducing soil erosion;^[^ ^74^ ^]^ however, these programs have received criticism for their use of a top‐down approach, limited access to capital, inadequate benefits, insecure land tenure, and insufficient community participation.^[^ ^128^ ^]^ To tackle these challenges, a collaborative research project was implemented in Ethiopia's Upper Blue Nile Basin. This originally interdisciplinary research and development project subsequently evolved into a transdisciplinary initiative during its implementation. This project was conducted as a component of the SATREPS project, the goal of which was to foster international research collaborations, in this case between Japanese and Ethiopian institutions (https://www.jst.go.jp/global/english/).

**Figure 5:**
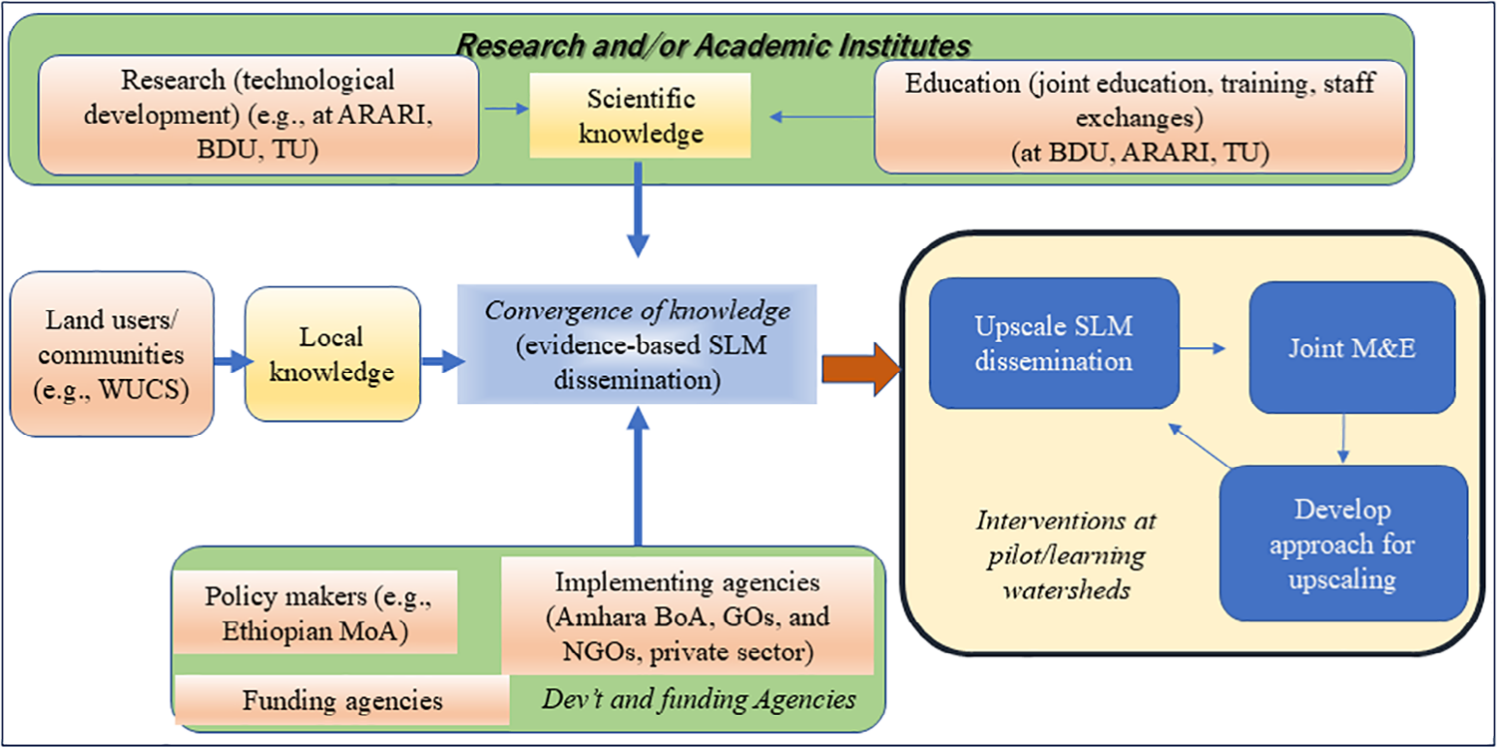
Workflow illustrating the implementation of a transdisciplinary partnership agreement in sustainable land‐use management (SLM) education and research activities. Key organizations and institutions include Amhara Regional Agricultural Research Institute (ARARI), Bahir Dar University (BDU), Tottori University (TU), Watershed Users Cooperative Society (WUCS), and Ministry of Agriculture (MoA). SLM, sustainable land management; M&E, monitoring, and evaluation; MoA, Ministry of Agriculture, GOs, governmental organizations; NGOs, nongovernmental organizations.

Aligned with the 15 year (2009–2023) Ethiopian Strategic Investment Framework, a national strategic planning framework prioritizing SLM investments,^[^ ^129^ ^]^ the aim of the SATREPS project in Ethiopia was to develop an evidence‐based and participatory SLM framework (https://www.jst.go.jp/global/english/kadai/h2801_ethiopia.html). The SLM framework incorporates various technologies that offer integrated benefits, including ecological improvements like reduced erosion and increased soil organic matter content, enhanced land productivity with improved crop and biomass yields, and access to socially and economically viable approaches, including participatory and socio‐economic empowerment strategies.^[^ ^86^ ^]^ By integrating these elements, the project has co‐created alternative watershed management scenarios,^[^ ^130^ ^]^ providing choices for land users and development organizations. A crucial output of this project is the establishment of a collaborative partnership involving researchers, policymakers, and local land users, creating an environment conducive to widespread adoption of evidence‐based SLM practices (**Figure** **5**).

Table S2 (Supporting Information) discusses the SATREPS project's features in the context of the proposed transdisciplinary research–development framework stages outlined in Figure 4. The SATREPS project in Ethiopia has created integrated SLM practices, disseminated its research findings in various forms including a guideline^[^ ^86^ ^]^ and policy brief,^[^ ^131^ ^]^ and received high evaluations for its impact and sustainability from its two funding agencies. One key driver for this success has been the coordinated implementation of three successive but initially independent project schemes that organically transformed into an interdependent structure, progressing from basic (KAKENHI Project, 2014–2018) to basic and applied (SATREPS Project, 2017–2022, and ultimately becoming development (Technical Cooperation Project, 2024–2028) projects (Table S2, Supporting Information). This model may serve as a reference for future long‐term projects aimed at addressing complex issues of land degradation and desertification.^[^ ^132^ ^]^

### 4.3 Limitations of the Study

To obtain a global picture of SLM research, policy, development practices, and erosion risk, we used several recognized country‐level data sources (including Scopus, Web of Science, WOCAT, FAOLEX, and the EU Soil Wiki). These sources served as provisionally useful proxies to obtain a global overview of science–policy–development interfaces. However, the partial nature of these proxies produces several limitations to this study.

First, only English‐language studies were used to ensure consistency in data interpretation and comparability, as translation limitations could introduce errors or misinterpretations. Additionally, most high‐impact environmental science research is published in English, which aligns with our study objective of capturing widely recognized scientific contributions. We acknowledge this as a limitation for its potential for selection bias.

Second, our research was constrained by the lack of available data on SLM dissemination for certain countries, particularly those classified as developed. These countries may have already implemented practices using their own resources but have not reported these practices to the WOCAT database which operates on a voluntary basis. The same limitation applies to the FAO database on policy and legal instruments. In developed countries, there is also a notable lack of diversity in implemented practices, policies, or research; instead, these countries tend to implement conservation agriculture that adheres to the three interlinked principles of minimum soil disturbance, biomass mulch soil cover, and crop diversification (e.g., Kassam et al., 2018).^[^ ^11^, ^133^ ^]^ Therefore, caution should be exercised when interpreting the number of SLM practices or policy documents in these countries.

Third, our study used the number of SLM papers, policies, and development practices as proxy indicators to evaluate the interfaces between science, policy, development practice, and soil erosion risk at the country level. This approach does not directly indicate the quality of these interfaces. For instance, a high number of published papers on SLM do not necessarily imply that they are of high quality or have policy or development relevance, and vice versa. Thus, our findings should be regarded as indicative and illustrative of global trends, rather than a detailed representation of the actual linkages in each specific country.

## 5 Conclusions and Implications for Future Studies

This paper offers a comprehensive, global‐scale assessment of SLM in the context of soil erosion risk and the degree to which each country is currently addressing this risk through evidence‐based SLM policies and implementation. Our analysis revealed that of the 236 countries assessed, 207 (88%) showed a low SPDI preparedness, 27 (11%) showed a moderate level of preparedness, and only 1 (0.4%; Argentina) showed a high level of preparedness to address the current soil erosion risk within cropland use categories. Strikingly, 109 countries (46%) face a high risk of soil erosion but have a low SPDI, while only 2 countries (0.9%; China and Argentina) boast high preparedness levels. The low SPDI is primarily attributed to low SDI, PDI, and SPI, in that order. This underscores the need to foster science‐based policy and development initiatives to mitigate the land degradation risk linked to soil erosion.

In response to these findings, we then proposed a transdisciplinary framework that bridges the gap between science, policy, and development by integrating evolving local and scientific knowledge. This approach incorporates diverse stakeholder perspectives into the development and dissemination of SLM practices. To enhance a weak SPDI, we recommend the following actions,

1. Reorient research to integrate scientific, societal, and environmental priorities.
2. Embrace iterative research processes that align with the research‐in‐development concept.
3. Translate research outcomes into practical tools and establish open‐access research evidence databases.
4. Enact evidence‐based policies essential for implementing research findings.
5. Foster participatory research‐extension linkages to ensure effective and widespread dissemination.
6. Implement objective ex‐post evaluations to extract lessons learned and enhance future projects.

The proposed transdisciplinary framework can improve the coordination between science, policy, and development sectors, enabling more tailored and effective strategies for combating soil erosion and promoting sustainable land management practices globally. Creating more effective collaborations between scientists, policymakers, and development agencies necessitates early engagement in the planning process. These measures collectively are expected to contribute to the creation of a more coherent and holistic approach to addressing soil erosion and land degradation through SLM.

Future research should focus on conducting long‐term studies to assess the effectiveness of (SLM) practices over time, particularly in underrepresented and vulnerable regions such as Sub‐Saharan Africa and Central Asia, where desertification and soil erosion are critical challenges. Additionally, efforts should explore the implementation of a transdisciplinary framework in countries with weak science–policy–development interfaces, not only by evaluating these interfaces but also by examining how the framework can be adapted and scaled to diverse national contexts through case studies in countries with varying policy, ecological, and socio‐economic conditions. Another key area for future research is the design of funding schemes that address gaps in science–policy–development interfaces, particularly in nations with limited financial resources for land management. This could involve assessing the effectiveness of different funding models in promoting transdisciplinary collaboration and ensuring the long‐term sustainability of SLM projects. Creating harmonized and accessible databases on science, policy, and development is the basis to addressing transdisciplinary environmental challenges like soil erosion.

## Conflict of Interest

The authors declare no conflict of interest.

## Supporting information



Supporting Information

## Data Availability

The data that support the findings of this study are available in the supplementary material of this article.
