# Supplementary material for: A Transdisciplinary Framework to Bridge Science–Policy–Development Gaps in Global Land Management Initiatives
Source: Glob Chall. 2025 May 22;9(8):2400261. doi: 10.1002/gch2.202400261 (PMC12371210; doi:10.1002/gch2.202400261)
Supplement: Supplementary file 1 — Supporting Information [file GCH2-9-2400261-s001.docx]

**Table S1.** Country level SLM practices, papers, policy documents, soil erosion risk in croplands subject to two or more erosion processes, science–policy interface (SPI), science–development interface (SDI), policy–development interface (PDI), and science–policy–development interface (SPDI).

| Name | #SLM practices | #SLM papers | #SLM policy | Cropland area (%) two or more erosion processes | SLM practices class | SLM papers class | SLM policy class | Erosion risk class | SPI | SDI | PDI | SPDI |
| --- | --- | --- | --- | --- | --- | --- | --- | --- | --- | --- | --- | --- |
| Afghanistan | 20 | 0 | 0 | 95 | Moderate | Low | Low | High | Low | Low | Low | Low |
| Aland | 0 | 0 | 0 | 34 | Low | Low | Low | Low | Low | Low | Low | Low |
| Albania | 1 | 0 | 5 | 94 | Low | Low | Low | High | Low | Low | Low | Low |
| Algeria | 0 | 0 | 3 | 98 | Low | Low | Low | High | Low | Low | Low | Low |
| American Samoa | 0 | 0 | 0 | 0 | Low | Low | Low | Low | Low | Low | Low | Low |
| Andorra | 0 | 0 | 0 | 90 | Low | Low | Low | High | Low | Low | Low | Low |
| Angola | 2 | 0 | 0 | 77 | Low | Low | Low | Moderate | Low | Low | Low | Low |
| Anguilla | 0 | 0 | 0 | 82 | Low | Low | Low | High | Low | Low | Low | Low |
| Antigua and Barbuda | 1 | 0 | 1 | 97 | Low | Low | Low | High | Low | Low | Low | Low |
| Argentina | 77 | 3 | 34 | 66 | High | Low | High | Moderate | Moderate | Moderate | High | High |
| Armenia | 2 | 0 | 5 | 94 | Low | Low | Low | High | Low | Low | Low | Low |
| Aruba | 0 | 0 | 0 | 52 | Low | Low | Low | Moderate | Low | Low | Low | Low |
| Ashmore and Cartier Islands | 0 | 0 | 0 | 0 | Low | Low | Low | Low | Low | Low | Low | Low |
| Australia | 6 | 0 | 37 | 51 | High | Low | High | Moderate | Moderate | Moderate | High | Moderate |
| Austria | 0 | 0 | 13 | 75 | Low | Low | Moderate | Moderate | Low | Low | Low | Low |
| Azerbaijan | 1 | 0 | 14 | 66 | Low | Low | Moderate | Moderate | Low | Low | Low | Low |
| Bahamas | 1 | 0 | 2 | 70 | Low | Low | Low | Moderate | Low | Low | Low | Low |
| Bahrain | 0 | 0 | 1 | 66 | Low | Low | Low | Moderate | Low | Low | Low | Low |
| Bangladesh | 19 | 0 | 0 | 99 | Moderate | Low | Low | High | Low | Low | Low | Low |
| Barbados |  | 0 | 0 | 99 | Low | Low | Low | High | Low | Low | Low | Low |
| Belarus | 1 | 0 | 7 | 3 | Low | Low | Moderate | Low | Low | Low | Low | Low |
| Belgium | 3 | 0 | 15 | 43 | Low | Low | Moderate | Moderate | Low | Low | Low | Low |
| Belize | 0 | 0 | 1 | 97 | Low | Low | Low | High | Low | Low | Low | Low |
| Benin | 1 | 0 | 1 | 67 | Low | Low | Low | Moderate | Low | Low | Low | Low |

**Table S1.** Continued.

| Name | #SLM practices | #SLM papers | #SLM policy | Cropland area (%) two or more erosion processes | SLM practices class | SLM papers class | SLM policy class | Erosion risk class | SPI | SDI | PDI | SPDI |
| --- | --- | --- | --- | --- | --- | --- | --- | --- | --- | --- | --- | --- |
| Bermuda | 0 | 0 | 0 | 0 | Low | Low | Low | Low | Low | Low | Low | Low |
| Bhutan | 10 | 0 | 1 | 100 | Low | Low | Low | High | Low | Low | Low | Low |
| Bolivia | 17 | 0 | 8 | 77 | Moderate | Low | Moderate | Moderate | Low | Low | Moderate | Moderate |
| Bosnia and Herzegovina | 3 | 0 | 2 | 91 | Low | Low | Low | High | Low | Low | Low | Low |
| Botswana | 5 | 0 | 2 | 65 | Low | Low | Low | Moderate | Low | Low | Low | Low |
| Brazil | 14 | 27 | 26 | 87 | High | Low | High | High | Moderate | Low | Moderate | Moderate |
| British Indian Ocean Territory | 0 | 0 | 0 | 0 | Low | Low | Low | Low | Low | Low | Low | Low |
| British Virgin Islands | 0 | 0 | 0 | 80 | Low | Low | Low | High | Low | Low | Low | Low |
| Brunei | 0 | 0 | 0 | 75 | Low | Low | Low | Moderate | Low | Low | Low | Low |
| Bulgaria | 6 | 0 | 7 | 86 | Low | Low | Moderate | High | Low | Low | Low | Low |
| Burkina Faso | 17 | 5 | 1 | 77 | Moderate | Low | Low | Moderate | Low | Low | Low | Low |
| Burundi | 12 | 2 | 4 | 97 | Low | Low | Low | High | Low | Low | Low | Low |
| Cambodia | 42 | 0 | 3 | 89 | Moderate | Low | Low | High | Low | Low | Low | Low |
| Cameroon | 5 | 0 | 2 | 74 | Low | Low | Low | Moderate | Low | Low | Low | Low |
| Canada | 2 | 20 | 22 | 76 | High | Low | Moderate | Moderate | Low | Moderate | Moderate | Moderate |
| Cape Verde | 10 | 2 | 12 | 0 | Low | Low | Moderate | Low | Low | Low | Low | Low |
| Cayman Islands | 0 | 0 | 0 | 67 | Low | Low | Low | Moderate | Low | Low | Low | Low |
| Central African Republic | 1 | 0 | 0 | 30 | Low | Low | Low | Low | Low | Low | Low | Low |
| Chad | 4 | 0 | 0 | 60 | Low | Low | Low | Moderate | Low | Low | Low | Low |
| Chile | 11 | 5 | 11 | 74 | Low | Low | Moderate | Moderate | Low | Low | Low | Low |
| China | 53 | 474 | 16 | 90 | High | High | Moderate | High | Moderate | High | Moderate | High |
| Cocos Islands | 0 | 0 | 0 | 0 | Low | Low | Low | Low | Low | Low | Low | Low |
| Colombia | 12 | 0 | 12 | 96 | Low | Low | Moderate | High | Low | Low | Low | Low |
| Comoros | 0 | 0 | 4 | 98 | Low | Low | Low | High | Low | Low | Low | Low |

**Table S1.** Continued.

| Name | #SLM practices | #SLM papers | #SLM policy | Cropland area (%) two or more erosion processes | SLM practices class | SLM papers class | SLM policy class | Erosion risk class | SPI | SDI | PDI | SPDI |
| --- | --- | --- | --- | --- | --- | --- | --- | --- | --- | --- | --- | --- |
| Congo | 1 | 0 | 2 | 84 | Low | Low | Low | High | Low | Low | Low | Low |
| Costa Rica | 4 | 0 | 9 | 99 | Low | Low | Moderate | High | Low | Low | Low | Low |
| Croatia | 0 | 0 | 6 | 71 | Low | Low | Low | Moderate | Low | Low | Low | Low |
| Cuba | 5 | 0 | 15 | 98 | Low | Low | Moderate | High | Low | Low | Low | Low |
| Curaçao | 0 | 0 | 0 | 97 | Low | Low | Low | High | Low | Low | Low | Low |
| Cyprus | 4 | 0 | 3 | 98 | Low | Low | Low | High | Low | Low | Low | Low |
| Czech Republic | 0 | 0 | 2 | 54 | Low | Low | Low | Moderate | Low | Low | Low | Low |
| Democratic Republic of the Congo | 2 | 0 | 3 | 86 | Low | Low | Low | High | Low | Low | Low | Low |
| Denmark | 0 | 0 | 8 | 21 | Low | Low | Moderate | Low | Low | Low | Low | Low |
| Djibouti | 1 | 0 | 1 | 100 | Low | Low | Low | High | Low | Low | Low | Low |
| Dominica | 1 | 0 | 0 | 98 | Low | Low | Low | High | Low | Low | Low | Low |
| Dominican Republic | 1 | 0 | 6 | 96 | Low | Low | Low | High | Low | Low | Low | Low |
| East Timor | 0 | 0 | 0 | 98 | Low | Low | Low | High | Low | Low | Low | Low |
| Ecuador | 13 | 1 | 7 | 93 | Moderate | Low | Moderate | High | Low | Low | Moderate | Moderate |
| Egypt | 1 | 0 | 4 | 58 | Low | Low | Low | Moderate | Low | Low | Low | Low |
| El Salvador | 2 | 0 | 4 | 100 | Low | Low | Low | High | Low | Low | Low | Low |
| Equatorial Guinea | 0 | 0 | 1 | 61 | Low | Low | Low | Moderate | Low | Low | Low | Low |
| Eritrea | 5 | 0 | 0 | 87 | Low | Low | Low | High | Low | Low | Low | Low |
| Estonia | 3 | 0 | 9 | 3 | Low | Low | Moderate | Low | Low | Low | Low | Low |
| eSwatini | 0 | 0 | 1 | 90 | Low | Low | Low | High | Low | Low | Low | Low |
| Ethiopia | 83 | 94 | 9 | 96 | High | Moderate | Moderate | High | Moderate | Moderate | Moderate | Moderate |
| Falkland Islands | 0 | 0 | 0 | 75 | Low | Low | Low | Moderate | Low | Low | Low | Low |
| Faroe Islands | 0 | 0 | 0 | 3 | Low | Low | Low | Low | Low | Low | Low | Low |
| Federated States of Micronesia | 0 | 0 | 0 | 0 | Low | Low | Low | Low | Low | Low | Low | Low |

**Table S1.** Continued.

| Name | #SLM practices | #SLM papers | #SLM policy | Cropland area (%) two or more erosion processes | SLM practices class | SLM papers class | SLM policy class | Erosion risk class | SPI | SDI | PDI | SPDI |
| --- | --- | --- | --- | --- | --- | --- | --- | --- | --- | --- | --- | --- |
| Fiji | 1 | 0 | 1 | 97 | Low | Low | Low | High | Low | Low | Low | Low |
| Finland | 0 | 0 | 6 | 25 | Low | Low | Low | Low | Low | Low | Low | Low |
| France | 14 | 13 | 4 | 49 | Moderate | Low | Low | Moderate | Low | Low | Low | Low |
| French Polynesia | 0 | 0 | 0 | 0 | Low | Low | Low | Low | Low | Low | Low | Low |
| French Southern and Antarctic Lands | 0 | 0 | 0 | 0 | Low | Low | Low | Low | Low | Low | Low | Low |
| Gabon | 3 | 0 | 4 | 80 | Low | Low | Low | High | Low | Low | Low | Low |
| Gambia | 0 | 0 | 1 | 47 | Low | Low | Low | Moderate | Low | Low | Low | Low |
| Georgia | 1 | 0 | 8 | 92 | Low | Low | Moderate | High | Low | Low | Low | Low |
| Germany | 14 | 30 | 7 | 32 | Moderate | Low | Moderate | Low | Low | Low | Moderate | Moderate |
| Ghana | 4 | 0 | 5 | 64 | Low | Low | Low | Moderate | Low | Low | Low | Low |
| Greece | 19 | 9 | 8 | 88 | Moderate | Low | Moderate | High | Low | Low | Moderate | Moderate |
| Greenland | 0 | 0 | 0 | 67 | Low | Low | Low | Moderate | Low | Low | Low | Low |
| Grenada | 0 | 0 | 4 | 97 | Low | Low | Low | High | Low | Low | Low | Low |
| Guam | 0 | 0 | 1 | 0 | Low | Low | Low | Low | Low | Low | Low | Low |
| Guatemala | 0 | 0 | 11 | 98 | Low | Low | Moderate | High | Low | Low | Low | Low |
| Guernsey | 0 | 0 | 0 | 83 | Low | Low | Low | High | Low | Low | Low | Low |
| Guinea | 3 | 0 | 2 | 88 | Low | Low | Low | High | Low | Low | Low | Low |
| Guinea-Bissau | 1 | 0 | 0 | 46 | Low | Low | Low | Moderate | Low | Low | Low | Low |
| Guyana | 1 | 0 | 3 | 58 | Low | Low | Low | Moderate | Low | Low | Low | Low |
| Haiti | 9 | 1 | 4 | 98 | Low | Low | Low | High | Low | Low | Low | Low |
| Heard Island and McDonald Islands | 0 | 0 | 0 | 0 | Low | Low | Low | Low | Low | Low | Low | Low |
| Honduras | 29 | 0 | 10 | 99 | Low | Low | Moderate | High | Low | Low | Low | Low |
| Hungary | 8 | 5 | 8 | 22 | Low | Low | Moderate | Low | Low | Low | Low | Low |
| Iceland | 4 | 0 | 1 | 72 | Low | Low | Low | Moderate | Low | Low | Low | Low |
| India | 60 | 56 | 3 | 85 | High | Moderate | Low | High | Low | Moderate | Moderate | Moderate |

**Table S1.** Continued.

| Name | #SLM practices | #SLM papers | #SLM policy | Cropland area (%) two or more erosion processes | SLM practices class | SLM papers class | SLM policy class | Erosion risk class | SPI | SDI | PDI | SPDI |
| --- | --- | --- | --- | --- | --- | --- | --- | --- | --- | --- | --- | --- |
| Indian Ocean Territories | 0 | 0 | 0 | 0 | Low | Low | Low | Low | Low | Low | Low | Low |
| Indonesia | 3 | 0 | 5 | 93 | Low | Low | Low | High | Low | Low | Low | Low |
| Iran | 5 | 0 | 5 | 97 | Low | Low | Low | High | Low | Low | Low | Low |
| Iraq | 0 | 0 | 1 | 96 | Low | Low | Low | High | Low | Low | Low | Low |
| Ireland | 0 | 0 | 7 | 43 | Low | Low | Moderate | Moderate | Low | Low | Low | Low |
| Isle of Man | 0 | 0 | 0 | 95 | Low | Low | Low | High | Low | Low | Low | Low |
| Israel | 0 | 0 | 0 | 99 | Low | Low | Low | High | Low | Low | Low | Low |
| Italy | 26 | 20 | 7 | 79 | Moderate | Low | Moderate | High | Low | Low | Moderate | Moderate |
| Ivory Coast | 1 | 0 | 5 | 47 | Low | Low | Low | Moderate | Low | Low | Low | Low |
| Jamaica | 1 | 0 | 10 | 100 | Low | Low | Moderate | High | Low | Low | Low | Low |
| Japan | 0 | 0 | 1 | 93 | Low | Low | Low | High | Low | Low | Low | Low |
| Jersey | 0 | 0 | 0 | 94 | Low | Low | Low | High | Low | Low | Low | Low |
| Jordan | 1 | 0 | 6 | 99 | Low | Low | Low | High | Low | Low | Low | Low |
| Kazakhstan | 18 | 0 | 2 | 63 | Moderate | Low | Low | Moderate | Low | Low | Low | Low |
| Kenya | 88 | 24 | 7 | 87 | High | Low | Moderate | High | Low | Moderate | Moderate | Moderate |
| Kiribati | 0 | 0 | 1 | 0 | Low | Low | Low | Low | Low | Low | Low | Low |
| Kosovo | 0 | 0 | 0 | 93 | Low | Low | Low | High | Low | Low | Low | Low |
| Kuwait | 23 | 0 | 0 | 73 | Moderate | Low | Low | Moderate | Low | Low | Low | Low |
| Kyrgyzstan | 15 | 0 | 3 | 94 | Moderate | Low | Low | High | Low | Low | Low | Low |
| Laos | 19 | 2 | 4 | 91 | Moderate | Low | Low | High | Low | Low | Low | Low |
| Latvia | 0 | 0 | 3 | 2 | Low | Low | Low | Low | Low | Low | Low | Low |
| Lebanon | 5 | 0 | 10 | 99 | Low | Low | Moderate | High | Low | Low | Low | Low |
| Lesotho | 1 | 0 | 3 | 100 | Low | Low | Low | High | Low | Low | Low | Low |
| Liberia | 0 | 0 | 3 | 51 | Low | Low | Low | Moderate | Low | Low | Low | Low |

**Table S1.** Continued.

| Name | #SLM practices | #SLM papers | #SLM policy | Cropland area (%) two or more erosion processes | SLM practices class | SLM papers class | SLM policy class | Erosion risk class | SPI | SDI | PDI | SPDI |
| --- | --- | --- | --- | --- | --- | --- | --- | --- | --- | --- | --- | --- |
| Libya | 1 | 0 | 0 | 89 | Low | Low | Low | High | Low | Low | Low | Low |
| Liechtenstein | 0 | 0 | 0 | 76 | Low | Low | Low | Moderate | Low | Low | Low | Low |
| Lithuania | 0 | 0 | 10 | 2 | Low | Low | Moderate | Low | Low | Low | Low | Low |
| Luxembourg | 0 | 0 | 2 | 69 | Low | Low | Low | Moderate | Low | Low | Low | Low |
| Macedonia | 1 | 0 | 0 | 93 | Low | Low | Low | High | Low | Low | Low | Low |
| Madagascar | 7 | 0 | 5 | 93 | Low | Low | Low | High | Low | Low | Low | Low |
| Malawi | 0 | 0 | 5 | 91 | Low | Low | Low | High | Low | Low | Low | Low |
| Malaysia | 0 | 0 | 2 | 76 | Low | Low | Low | Moderate | Low | Low | Low | Low |
| Maldives | 0 | 0 | 1 | 0 | Low | Low | Low | Low | Low | Low | Low | Low |
| Mali | 46 | 2 | 0 | 79 | Moderate | Low | Low | High | Low | Low | Low | Low |
| Malta | 0 | 0 | 3 | 99 | Low | Low | Low | High | Low | Low | Low | Low |
| Marshall Islands | 0 | 0 | 2 | 0 | Low | Low | Low | Low | Low | Low | Low | Low |
| Mauritania | 3 | 0 | 0 | 94 | Low | Low | Low | High | Low | Low | Low | Low |
| Mauritius | 0 | 0 | 4 | 95 | Low | Low | Low | High | Low | Low | Low | Low |
| Mexico | 24 | 3 | 53 | 93 | Low | Low | High | High | Moderate | Low | Moderate | Moderate |
| Moldova | 5 | 0 | 5 | 93 | Low | Low | Low | High | Low | Low | Low | Low |
| Monaco | 0 | 0 | 0 | 33 | Low | Low | Low | Low | Low | Low | Low | Low |
| Mongolia | 5 | 0 | 2 | 94 | Low | Low | Low | High | Low | Low | Low | Low |
| Montenegro | 0 | 0 | 0 | 99 | Low | Low | Low | High | Low | Low | Low | Low |
| Montserrat | 0 | 0 | 0 | 86 | Low | Low | Low | High | Low | Low | Low | Low |
| Morocco | 50 | 1 | 4 | 99 | High | Low | Low | High | Low | Moderate | Moderate | Moderate |
| Mozambique | 0 | 0 | 0 | 68 | Low | Low | Low | Moderate | Low | Low | Low | Low |
| Myanmar | 0 | 0 | 2 | 91 | Low | Low | Low | High | Low | Low | Low | Low |
| Namibia | 10 | 0 | 2 | 73 | Low | Low | Low | Moderate | Low | Low | Low | Low |

**Table S1.** Continued.

| Name | #SLM practices | #SLM papers | #SLM policy | Cropland area (%) two or more erosion processes | SLM practices class | SLM papers class | SLM policy class | Erosion risk class | SPI | SDI | PDI | SPDI |
| --- | --- | --- | --- | --- | --- | --- | --- | --- | --- | --- | --- | --- |
| Nauru | 0 | 0 | 1 | 0 | Low | Low | Low | Low | Low | Low | Low | Low |
| Nepal | 86 | 1 | 4 | 99 | High | Low | Low | High | Low | Moderate | Moderate | Moderate |
| Netherlands | 12 | 72 | 3 | 10 | Low | Moderate | Low | Low | Low | Low | Low | Low |
| New Caledonia | 0 | 0 | 0 | 98 | Low | Low | Low | High | Low | Low | Low | Low |
| New Zealand | 0 | 0 | 2 | 88 | High | Low | Low | High | Low | Moderate | Moderate | Moderate |
| Nicaragua | 16 | 0 | 4 | 100 | Low | Low | Low | High | Low | Low | Low | Low |
| Niger | 58 | 1 | 1 | 97 | High | Low | Low | High | Low | Moderate | Moderate | Moderate |
| Nigeria | 0 | 0 | 0 | 76 | Low | Low | Low | Moderate | Low | Low | Low | Low |
| Niue | 0 | 0 | 0 | 0 | Low | Low | Low | Low | Low | Low | Low | Low |
| Norfolk Island | 0 | 0 | 0 | 0 | Low | Low | Low | Low | Low | Low | Low | Low |
| North Korea | 0 | 0 | 0 | 99 | Low | Low | Low | High | Low | Low | Low | Low |
| Northern Cyprus | 0 | 0 | 0 | 95 | Low | Low | Low | High | Low | Low | Low | Low |
| Northern Mariana Islands | 0 | 0 | 0 | 0 | Low | Low | Low | Low | Low | Low | Low | Low |
| Norway | 3 | 0 | 2 | 78 | Low | Low | Low | Moderate | Low | Low | Low | Low |
| Oman | 0 | 0 | 2 | 94 | Low | Low | Low | High | Low | Low | Low | Low |
| Pakistan | 8 | 6 | 1 | 52 | Low | Low | Low | Moderate | Low | Low | Low | Low |
| Palau | 1 | 0 | 0 | 25 | Low | Low | Low | Low | Low | Low | Low | Low |
| Palestine | 0 | 0 | 0 | 100 | Low | Low | Low | High | Low | Low | Low | Low |
| Panama | 0 | 0 | 8 | 99 | Low | Low | Moderate | High | Low | Low | Low | Low |
| Papua New Guinea | 0 | 0 | 0 | 89 | Low | Low | Low | High | Low | Low | Low | Low |
| Paraguay | 0 | 0 | 9 | 78 | Low | Low | Moderate | Moderate | Low | Low | Low | Low |
| Peru | 16 | 2 | 20 | 96 | Moderate | Low | Moderate | High | Low | Low | Moderate | Moderate |
| Philippines | 51 | 2 | 4 | 98 | High | Low | Low | High | Low | Moderate | Moderate | Moderate |
| Pitcairn Islands | 0 | 0 | 0 | 0 | Low | Low | Low | Low | Low | Low | Low | Low |

**Table S1.** Continued.

| Name | #SLM practices | #SLM papers | #SLM policy | Cropland area (%) two or more erosion processes | SLM practices class | SLM papers class | SLM policy class | Erosion risk class | SPI | SDI | PDI | SPDI |
| --- | --- | --- | --- | --- | --- | --- | --- | --- | --- | --- | --- | --- |
| Poland | 5 | 2 | 4 | 11 | Low | Low | Low | Low | Low | Low | Low | Low |
| Portugal | 8 | 0 | 17 | 91 | Low | Low | Moderate | High | Low | Low | Low | Low |
| Puerto Rico | 0 | 0 | 0 | 99 | Low | Low | Low | High | Low | Low | Low | Low |
| Qatar | 0 | 0 | 1 | 34 | Low | Low | Low | Low | Low | Low | Low | Low |
| Republic of Serbia | 1 | 0 | 3 | 59 | Low | Low | Low | Moderate | Low | Low | Low | Low |
| Romania | 6 | 0 | 4 | 67 | Low | Low | Low | Moderate | Low | Low | Low | Low |
| Russia | 17 | 2 | 31 | 63 | Moderate | Low | High | Moderate | Moderate | Low | Moderate | Moderate |
| Rwanda | 8 | 0 | 3 | 96 | Low | Low | Low | High | Low | Low | Low | Low |
| Saint Barthelemy | 0 | 0 | 0 | 92 | Low | Low | Low | High | Low | Low | Low | Low |
| Saint Helena | 0 | 0 | 0 | 75 | Low | Low | Low | Moderate | Low | Low | Low | Low |
| Saint Kitts and Nevis | 1 | 0 | 1 | 0 | Low | Low | Low | Low | Low | Low | Low | Low |
| Saint Lucia | 2 | 0 | 4 | 98 | Low | Low | Low | High | Low | Low | Low | Low |
| Saint Martin | 0 | 0 | 0 | 97 | Low | Low | Low | High | Low | Low | Low | Low |
| Saint Pierre and Miquelon | 0 | 0 | 0 | 94 | Low | Low | Low | High | Low | Low | Low | Low |
| Saint Vincent and the Grenadines | 0 | 0 | 5 | 17 | Low | Low | Low | Low | Low | Low | Low | Low |
| Samoa | 0 | 0 | 2 | 96 | Low | Low | Low | High | Low | Low | Low | Low |
| San Marino | 0 | 0 | 0 | 0 | Low | Low | Low | Low | Low | Low | Low | Low |
| Sao Tome and Principe | 0 | 0 | 0 | 99 | Low | Low | Low | High | Low | Low | Low | Low |
| Saudi Arabia | 1 | 0 | 1 | 86 | Low | Low | Low | High | Low | Low | Low | Low |
| Senegal | 45 | 0 | 3 | 49 | Moderate | Low | Low | Moderate | Low | Low | Low | Low |
| Seychelles | 0 | 0 | 1 | 0 | Low | Low | Low | Low | Low | Low | Low | Low |
| Sierra Leone | 0 | 0 | 2 | 51 | Low | Low | Low | Moderate | Low | Low | Low | Low |
| Singapore | 0 | 0 | 0 | 85 | Low | Low | Low | High | Low | Low | Low | Low |
| Sint Maarten | 0 | 0 | 0 | 90 | Low | Low | Low | High | Low | Low | Low | Low |

**Table S1.** Continued.

| Name | #SLM practices | #SLM papers | #SLM policy | Cropland area (%) two or more erosion processes | SLM practices class | SLM papers class | SLM policy class | Erosion risk class | SPI | SDI | PDI | SPDI |
| --- | --- | --- | --- | --- | --- | --- | --- | --- | --- | --- | --- | --- |
| Slovakia | 4 | 0 | 0 | 69 | Low | Low | Low | Moderate | Low | Low | Low | Low |
| Slovenia | 4 | 0 | 2 | 91 | Low | Low | Low | High | Low | Low | Low | Low |
| Solomon Islands | 0 | 0 | 1 | 99 | Low | Low | Low | High | Low | Low | Low | Low |
| Somalia | 0 | 0 | 0 | 88 | Low | Low | Low | High | Low | Low | Low | Low |
| South Africa | 53 | 4 | 5 | 85 | High | Low | Low | High | Low | Moderate | Moderate | Moderate |
| South Georgia and the Islands | 0 | 0 | 0 | 0 | Low | Low | Low | Low | Low | Low | Low | Low |
| South Korea | 0 | 0 | 7 | 100 | Low | Low | Moderate | High | Low | Low | Low | Low |
| South Sudan | 0 | 0 | 0 | 26 | Low | Low | Low | Low | Low | Low | Low | Low |
| Spain | 36 | 40 | 3 | 95 | Moderate | Low | Low | High | Low | Low | Low | Low |
| Sri Lanka | 2 | 0 | 1 | 79 | Low | Low | Low | Moderate | Low | Low | Low | Low |
| Sudan | 4 | 0 | 0 | 78 | Low | Low | Low | Moderate | Low | Low | Low | Low |
| Suriname | 1 | 0 | 0 | 58 | Low | Low | Low | Moderate | Low | Low | Low | Low |
| Sweden | 2 | 0 | 2 | 15 | Low | Low | Low | Low | Low | Low | Low | Low |
| Switzerland | 23 | 11 | 9 | 98 | Moderate | Low | Moderate | High | Low | Low | Moderate | Moderate |
| Syria | 8 | 0 | 1 | 95 | Low | Low | Low | High | Low | Low | Low | Low |
| Taiwan | 0 | 0 | 0 | 100 | Low | Low | Low | High | Low | Low | Low | Low |
| Tajikistan | 169 | 1 | 1 | 95 | High | Low | Low | High | Low | Moderate | Moderate | Moderate |
| Tanzania | 43 | 13 | 3 | 86 | Moderate | Low | Low | High | Low | Low | Low | Low |
| Thailand | 14 | 0 | 2 | 85 | Moderate | Low | Low | High | Low | Low | Low | Low |
| Togo | 11 | 0 | 2 | 61 | Low | Low | Low | Moderate | Low | Low | Low | Low |
| Tonga | 2 | 0 | 0 | 34 | Low | Low | Low | Low | Low | Low | Low | Low |
| Trinidad and Tobago | 0 | 0 | 4 | 99 | Low | Low | Low | High | Low | Low | Low | Low |
| Tunisia | 35 | 5 | 4 | 97 | Moderate | Low | Low | High | Low | Low | Low | Low |
| Turkey | 9 | 4 | 15 | 94 | Low | Low | Moderate | High | Low | Low | Low | Low |
| Turkmenistan | 3 | 0 | 0 | 97 | Low | Low | Low | High | Low | Low | Low | Low |

**Table S1.** Continued.

| Name | #SLM practices | | #SLM papers | | #SLM policy | | Cropland area (%) two or more erosion processes | | SLM practices class | | SLM papers class | | SLM policy class | | Erosion risk class | | SPI | | SDI | | PDI | | SPDI | |
| --- | --- | --- | --- | --- | --- | --- | --- | --- | --- | --- | --- | --- | --- | --- | --- | --- | --- | --- | --- | --- | --- | --- | --- | --- |
| Turks and Caicos Islands | 0 | 0 | | 0 | | 95 | | Low | | Low | | Low | | High | | Low | | Low | | Low | | Low | |  |
| Uganda | 68 | 6 | | 9 | | 86 | | High | | Low | | Moderate | | High | | Low | | Moderate | | Moderate | | Moderate | |  |
| Ukraine | 2 | 0 | | 43 | | 53 | | Low | | Low | | High | | Moderate | | Moderate | | Low | | Moderate | | Moderate | |  |
| United Arab Emirates | 2 | 0 | | 1 | | 77 | | Low | | Low | | Low | | Moderate | | Low | | Low | | Low | | Low | |  |
| United Kingdom | 13 | 39 | | 12 | | 55 | | Moderate | | Moderate | | Moderate | | Moderate | | Moderate | | Moderate | | Moderate | | Moderate | |  |
| United States of America | 1 | 141 | | 14 | | 87 | | High | | Moderate | | Moderate | | High | | Moderate | | Moderate | | Moderate | | Moderate | |  |
| United States Virgin Islands | 0 | 0 | | 0 | | 50 | | Low | | Low | | Low | | Moderate | | Low | | Low | | Low | | Low | |  |
| Uruguay | 0 | 0 | | 9 | | 94 | | Low | | Low | | Moderate | | High | | Low | | Low | | Low | | Low | |  |
| Uzbekistan | 26 | 0 | | 13 | | 83 | | Moderate | | Low | | Moderate | | High | | Low | | Low | | Moderate | | Moderate | |  |
| Vanuatu | 0 | 0 | | 1 | | 100 | | Low | | Low | | Low | | High | | Low | | Low | | Low | | Low | |  |
| Vatican | 0 | 0 | | 0 | | 0 | | Low | | Low | | Low | | Low | | Low | | Low | | Low | | Low | |  |
| Venezuela | 0 | 0 | | 15 | | 85 | | Low | | Low | | Moderate | | High | | Low | | Low | | Low | | Low | |  |
| Vietnam | 5 | 0 | | 6 | | 98 | | Low | | Low | | Low | | High | | Low | | Low | | Low | | Low | |  |
| Wallis and Futuna | 0 | 0 | | 0 | | 0 | | Low | | Low | | Low | | Low | | Low | | Low | | Low | | Low | |  |
| Western Sahara | 0 | 0 | | 0 | | 84 | | Low | | Low | | Low | | High | | Low | | Low | | Low | | Low | |  |
| Yemen | 9 | 0 | | 3 | | 99 | | Low | | Low | | Low | | High | | Low | | Low | | Low | | Low | |  |
| Zambia | 5 | 0 | | 1 | | 90 | | Low | | Low | | Low | | High | | Low | | Low | | Low | | Low | |  |
| Zimbabwe | 1 | 0 | | 0 | | 90 | | Low | | Low | | Low | | High | | Low | | Low | | Low | | Low | |  |

| Project cycles | Descriptions |
| --- | --- |
| Shared research framing | Problems were identified via field visits, prior research, and stakeholder discussions, aligning with policy and funding agency’s requirements. The project members involved over 30 interdisciplinary team of Japanese and Ethiopian researchers from four academic/research institutes and two development organizations. Collaborative framing produced shared research objectives, indicators, and implementation plans. However, land users' input was indirectly, acknowledged through surveys and meetings. |
| Research grant acquisition | The SATREPS project, spanning 3-5 years, follows a rigorous process including ODA (Official Development Assistance) requests, proposal submissions, reviews, and collaborative agreements for implementation. With a budget of 500 million JPY, funded by JST and JICA, it aims to bridge basic and applied research.  Succeeding coincidentally a previous project in the same basin (2014-2018), it builds on valuable basic research results and data and groundwork (<https://www.jsps.go.jp/english/e-grants/grants01.html>.)  JICA plans to support dissemination of SATREPS results (2024-2028), allocating 300 million JPY for upscaling sustainable land management. This succession of projects, facilitated by common core researchers, highlights the need for comprehensive, long-term funding programs to transition from research to practical applications effectively. |
| Co-creation of integrated practices | The project devised and executed five integrated outputs, focusing on soil erosion reduction, enhanced land productivity, income generation, and community participation. Testing 43 technologies and 4 approaches in Upper Blue Nile Basin, the best selected ones were validated under farmer-managed conditions across different agroecologies. Selected thirteen technologies and four approaches (Tsunekawa et al., 2023) are currently being adopted by development partners in the region. |
| SLM guidelines and policy brief | Produced 80 scientific articles and developed an SLM guideline book covering 13 technologies and approaches, along with policy brief documents. These resources offer practical guidance for implementing sustainable land management practices. |
| Research communication | The project disseminated its findings through various channels, with about 80% of scientific papers available via open access (<https://www.alrc.tottori-u.ac.jp/slm/performance/publication_papers.pdf> ). Results were presented at 42 conferences, annual stakeholders field days. Online presence was maintained through dedicated websites and social media channels. Workshops, meetings, and joint coordination committees were held regularly, alongside documentary videos and publications accessible through open access platforms. |
| Approach developed for evidence-based SLM dissemination | A Regional Research-Based Sustainable Land Management Partnership was formed to promote evidence-based SLM practices, using a transdisciplinary approach to tackle land degradation (Fig. 5). It engages diverse stakeholders and focuses on verifying technologies, disseminating methodologies, allocating resources, addressing field challenges through research, and implementing and monitoring science-based activities in model watersheds. |
| Project evaluation | The project underwent both midterm and terminal evaluations independently by JICA and JST. From JICA's perspective, the project received high ratings for relevance, alignment with global policy and development frameworks, effectiveness, efficiency, impact, and sustainability (JICA, 2023). Whereas an external expert evaluation by JST resulted in an "S" rating, indicating that the project not only met but exceeded its planned objectives (<https://www.alrc.tottori-u.ac.jp/english/news-detail.php?id=45> ). |

Table S2. SATREPS project’s experience from Ethiopia in view of the seven major components of the proposed transdisciplinary framework.
